# Engineered myoglobin as a catalyst for atom transfer radical cyclisation†

**DOI:** 10.1039/d2cc03227a

**Published:** 2022-08-31

**Authors:** Andriy Lubskyy, Chao Guo, Robert J. Chadwick, Alke Petri-Fink, Nico Bruns, Michela M. Pellizzoni

**Affiliations:** Adolphe Merkle Institute, University of Fribourg, Chemin des Verdiers 4,1700 Fribourg Switzerland michela.pellizzoni@unif.ch; Department of Pure and Applied Chemistry, University of Strathclyde 295 Cathedral Street Glasgow G1 1XL UK nico.bruns@tu-darmstadt.de; Department of Chemistry, University of Fribourg, Chemin du Musée 9,1700 Fribourg Switzerland; Department of Chemistry, Technical University of Darmstadt, Alarich-Weiss-Straße 4 64287 Darmstadt Germany

## Abstract

Myoglobin was subjected to site-directed mutagenesis and transformed into a catalyst able to perform atom transfer radical cyclisation reactions, *i.e*. intramolecular atom transfer radical additions. Replacing the iron-coordinating histidine with serine, or introducing small changes inside or at the entrance of the active site, transformed the completely inactive wild-type myoglobin into an artificial metalloenzyme able to catalyse the 5-*exo* cyclisation of halogenated unsaturated compounds for the synthesis of γ-lactams. This new-to-nature activity was achieved not only with purified protein but also in crude cell lysate and in whole cells.

## Abstract 2

Myoglobin was subjected to site-directed mutagenesis and transformed into a catalyst able to perform the atom transfer radical reaction.

## Graphical abstract

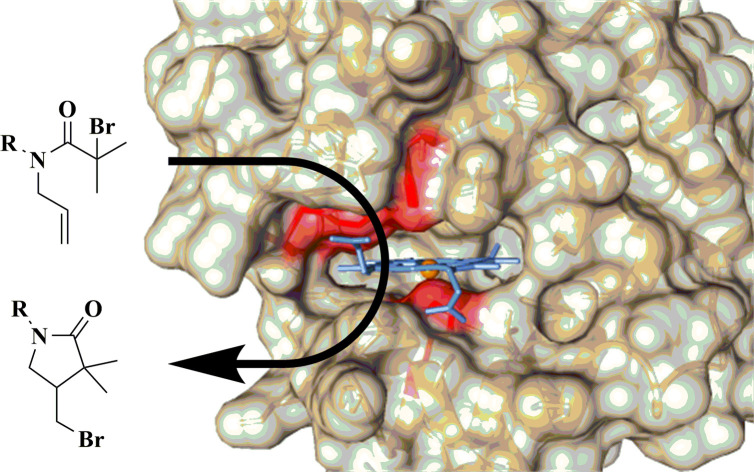

Over millions of years of evolution, nature developed and optimised its enzymatic toolbox to cover a great deal of chemical reactions for all her needs. Nevertheless, in the last century, chemists managed to dramatically expand the chemical reaction space, especially by utilising the properties of transition metals to develop catalysts for reactions that were not seen in nature before.^1^ In the past decades, innovations in fields of chemistry and molecular biology allowed to bridge the two fields, combining the novelty of human-made catalysts with the efficiency of enzymes.^2^ The pioneering work by Arnold demonstrated that by using mutagenesis and directed evolution, cytochromes can be modified to catalyse new-to-nature reactions such as carbene transfer,^3^ and consequently inspired a lot of research into unnatural enzymatic functionality.^4^

One of the examples of transition metal catalysed chemical transformations discovered in the lab is atom transfer radical addition (ATRA) that was first reported by Kharasch in the 1940s.^5^ It is a reaction of halogenated compounds with unsaturated carbon–carbon bonds, often catalysed by transition metal complexes.^6^ The intramolecular version of ATRA, atom transfer radical cyclisation (ATRC), gained interest within the organic chemistry community due to its ability to create cyclic molecules with 100% atom economy. It has been used in the synthesis of several natural products and pharmaceuticals, such as botryodiplodin or tyromycin A.^7^ Atom transfer radical reactions consist of several crucial mechanistic steps, mainly the abstraction of a halogen from a carbon atom catalysed by a transition metal complex in the reduced state, resulting in a carbon-centred radical. This subsequently reacts with an olefin double bond, resulting in a new C–C bond and another organic radical (Fig. 1A). This organic radical can abstract the halogen atom from the transition metal complex to form a new halogenated molecule in case of ATRC and ATRA, or further react with an alkene in case of the mechanistically related atom transfer radical polymerizations (ATRP). In the end, a new σ carbon–carbon bond is formed for every broken π carbon–carbon bond.

**Fig. 1:**
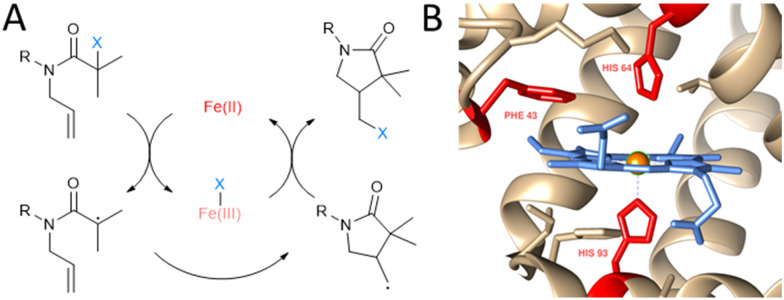
Non-natural ATRC catalytic activity of myoglobin mutants for the synthesis of γ-lactams. (A) Reaction scheme of iron catalysed ATRC. (B) Close up view of myoglobin active site (PDB: 1WLA) visualised with Chimera.^8^ The heme cofactor (blue), iron metal centre (orange) and residues Phe43, His64 and His93 (red) are highlighted. These amino acids were subjected to site directed mutagenesis in this study to achieve ATRC activity.

We and others have previously reported that iron and copper containing metalloenzymes such as peroxidases, haemoglobin, and laccases are capable of catalysing ATRP.^9^ Because of many similarities between ATRP and ATRC we hypothesised that ATRC can also be catalysed by heme proteins with peroxidase activity such as myoglobin, the monomeric version of haemoglobin known to have ATRPases activity. Confirmation that heme-containing protein can catalyse ATRC reaction was demonstrated by a recent work of Yang and coworkers.^4*d*^ They reported ATRC activity of engineered Cytochrome P450's using directed evolution.

Myoglobin (Mb; Fig. 1B) was chosen to be a starting point in the development of ATRCase. Myoglobin is a small globular oxygen carrier protein whose physiological properties have been extensively studied.^10^ Furthermore, with its ease of handling and engineering, its well-known structure and the ability to be easily expressed in bacterial systems, myoglobin is an excellent scaffold for the development of metalloenzymes with new-to-nature reactivity.^11^ In fact, its functionality was previously expanded to peroxidase,^12^ hydroxylase,^13^ and carbene transferase^14^ activity using mutagenesis, cofactor reconstitution and combination of both engineering methods.

Here, we report the first example of ATRC catalysis by engineered myoglobin. By introducing a serine mutation into a heme coordinating residue we produced a myoglobin variant that is capable of catalysing a range of bromine and chlorine transfers. This activity was obtained with purified protein as well as in cell lysate and with whole cell catalysis.

Cyclisation of *N*-allyl-alpha-haloamide 1 into gamma-lactam 2, a typical model reaction for the study of ATRC,^15^ was selected as a reaction to evaluate the ability of myoglobin to catalyse ATRC. The substrate consists of an α-halo bromide moiety with two methyl groups that stabilise the radical and a double bond that would lead to 5-*exo* cyclisation. Moreover, the benzyl group introduces constraints to the rotational axis of the amide bond, bringing the radical and alkene together. Reaction conditions included sodium ascorbate as a reducing agent to reduce any metmyoglobin (myoglobin in its Fe(iii) reduction state) to the active catalyst myoglobin in its Fe(ii) state at the beginning of the reaction, and to regenerate any metmyoglobin that might accumulate during the reaction into active Fe(ii) myoglobin similar to the well-established Activators ReGenerated by Electron Transfer (ARGET) method of conventional and biocatalytic ATRP.^16^ Studies with commercially available native myoglobin from equine skeleton muscle (Sigma-Aldrich, Germany) were unsuccessful in yielding cyclized product (Fig. 2). Therefore, a small library of single and double mutants was produced, targeting the iron axial ligand (His93) and the amino acids that surround the heme cofactor such as Phe43 and His64 (Fig. 1B and 2). Sperm whale myoglobin scaffold was used for the expression of recombinant myoglobin variants due to its 88% homology with commercial protein and similar folding behaviour.^17^ The mutations induced ATRC activity in myoglobin and the reactions yielded the halogenated lactam 2 in a 5-*exo-trig* cyclization, as evidenced by comparing the gas chromatography retention times and the mass spectra of reaction product and standard. Out of all variants with mutation in the axial ligand that exhibited ATRC activity, H93S was the best performing mutant with the total turnover number (TTN) of 62 (Fig. 2). We hypothesised that the first step of ATRC is associated with electron transfer from the ferrous iron of heme to the halogen atom of the organic substrate during halogen atom transfer from the substrate to the biocatalyst. A mutation of the amino acid that coordinates the metal will affect the redox potential of the metal.^10^ Every tested H93 mutant exhibited a redox potential much lower than wild type myoglobin (Fig. S1, ESI†), suggesting that high redox potential of the WT variant (+40 mv *vs.* SHE) could be unfavourable for electron transfer and initiation of ATRC. However, no direct correlation between the redox potential of myoglobin mutants and their ATRC activity were found. Despite its low redox potential, variant H93C (−186 mV *vs.* SHE) does not exhibit higher activity than variant H93S (−84 mV *vs.* SHE). Variants H93A (−87 mV *vs.* SHE) and H93S have similar redox potentials, but the latter resulted in twice as high turnover than the former. This catalytic behaviour indicates that in addition to the redox potential, polarity is another important parameter that affects electron transfer as reported by Matyjaszewski and co-workers for ATRP.^18^ However, we cannot exclude other reasons such as cofactor conformation in the binding site. This issue should be addressed in more details in next publications.

**Fig. 2:**
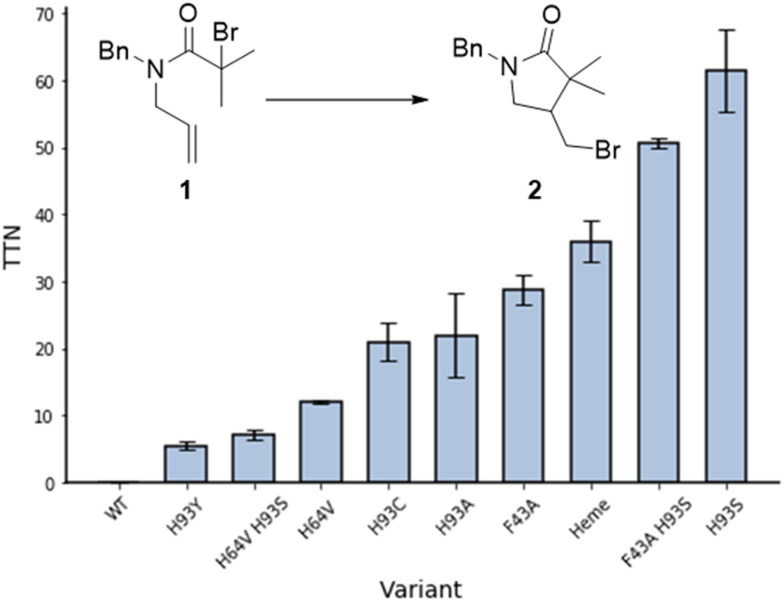
ATRC activity of myoglobin mutants for the production of γ-lactam 2. Reaction conditions: 30 mM 1, 0.1 mol% catalyst, 5 eq. sodium ascorbate, 40 °C, 16 h, PBS-Br (10 mM sodium phosphates, 100 mM NaBr, pH 7.4, 50 mM sodium phosphate/250 mM NaCl for reaction with heme), 3 vol% DMSO, inert atmosphere. TTN – total turnover numbers, average from 2 repetitions.

In order to obtain additional information on enzymatic ATRC, we investigated if other factors affect this new-to-nature activity. Replacing the bulky phenylalanine F43 at the entrance of the active site with a less space-constraining alanine frees up space around the metal centre, which should allow the substrate to more easily access the active site. The mutant F43A showed a turnover number up to 30, which supports this hypothesis (Fig. 2). Unfortunately, the combination of this mutation with the most potent axial mutation (F43A H93S) did not promote a synergistic effect in ATRC activity as the turn over number of the double mutant was similar to the turn over number of the single mutant H93S.

Another variant, H64V, resulted in increased activity compared to the wild type Mb (Fig. 2), potentially through freeing some space in the binding pocket. Replacing polar histidine with hydrophobic valine also affects the polarity of the binding pocket. However, the double mutant H64V H93S resulted in a lower turnover than the single mutant H64V.

In order to confirm the presence of the radical intermediate during the cyclisation reaction catalysed by myoglobin, the ATRC substrate 3*N*-benzyl-2-bromo-*N*-(3-cyclopropylallyl)-2-methylpropanamide was synthesised. This substrate contains an alkene substituted with a cyclopropyl moiety (Scheme 1), which will result in a ring opening reaction if a radical is in proximity.^19^ Production of the gamma lactam 4 by the H93S variant was detected (Scheme 1 and Fig. S2, ESI†), proving that the myoglobin catalysed cyclisation occurred through a radical mechanism.

**Scheme 1:**
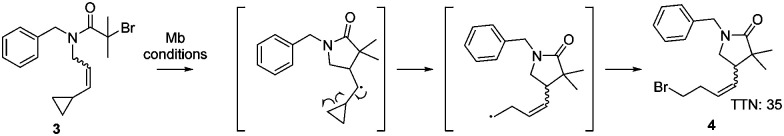
Experiment to confirm the radical mechanism in the ATRC reaction catalysed by Mb H93S. Reaction conditions: 30 mM 3, 0.1 mol% catalyst, 5 eq. sodium ascorbate, 40 °C, 16 h, phosphate buffer (50 mM, 250 mM NaCl, pH 7.4), 3 vol% DMSO, inert atmosphere.

In order to better understand the reaction behaviour and establish the critical reaction parameters, several control experiments were performed with the best performing myoglobin mutant H93S and the substrate 1. No product was formed in the absence of sodium ascorbate (Table S1, ESI,† entry 2), indicating the importance of the reducing agent for the activation of the catalyst and its regeneration in ARGET ATRC. Open flask conditions also did not result in the formation of product (Table S1, ESI,† entry 3), possibly due to high affinity of myoglobin towards oxygen or the quenching of the radical reaction by oxygen. No ATRC product was observed in the absence of myoglobin (Table S1, ESI,† entry 5), which proves that the ATRC was catalysed by the myoglobin mutants. The cofactor alone was also investigated for its ATRC activity and found to be approximately two-fold less effective than the H93S mutant (Table S1, ESI,† entry 6). Thus, while haem itself is an ATRC catalyst, the engineered H93S myoglobin mutant is more active. The stability of the final product in aqueous media was confirmed by incubation of compound 2 in buffer containing sodium ascorbate and myoglobin (Fig. S3, ESI†).

Having in hand an enzyme that is able to catalyse the new-to-nature ATRC reaction, we decided to investigate the possibility to use the mutant H93S in bacterial cell lysate and in *E. coli* whole cell catalysis (Table 1 entry 1 and Table S2, ESI†). This would omit protein purification steps during catalyst preparation, thereby simplifying the overall process, and because these reaction conditions could help to regenerate the catalyst during the reaction. It is well known that biocatalytic reactions can benefit from the whole cell machinery especially for cofactor recycling and because of the reductive environment within cells and cell lysate.^20^ In the presence of glucose as nutrient to activate the cellular metabolism, the total turnover number of 1 in whole cell and cell lysate experiments was 1.65- and 1.76-times higher than the TTN achieved with purified protein (Table 1, entry 1 and Table S2, ESI,† entries 1 and 7). We ruled out the possibility that glucose could act as reductant for myoglobin H93S (Table S2, ESI,† entry 4). Control experiments with *E.Coli* Dh5Alpha strain that does not express myoglobin showed significantly reduced ATRC activity. The residual activity could be attributed to natural cellular redox processes of residual traces of the cofactor (Table S3, ESI†). It is beyond the scope of this first report on myoglobin-catalysed ATRC to elucidate the details of whole cell ATRC biocatalysis. Therefore, it remains unknown which processes contribute to the enhanced turnover with cell lysate and in whole cell reactions. A possibility is that bacterial enzymes use glucose to produce NADH that can act as a reducing agent for myoglobin.^21^

**Table 1:** Substrate scope experiments for ATRC catalysed by Mb H93S as purified protein and in whole bacterial cells.

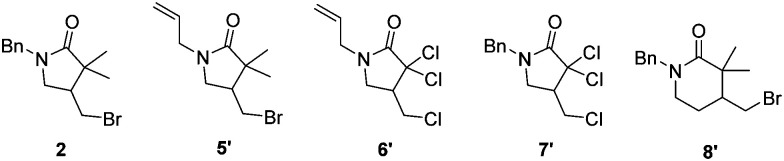			
Entry	Substrate/product	TTN (protein)^a^	TTN (whole cell)^b^
1	1/2	62 ± 6	101 ± 13
2	5/5′	4 ± 0	44 ± 5
3	6/6′	25 ± 2	28 ± 4
4	7/7′	22 ± 1	24 ± 1
5	8/8′	7 ± 6	18 ± 1

a Reaction conditions: 30 mM substrate, 0.1 mol% Mb H93S, 5 eq. sodium ascorbate, 40 °C, 16 h, sodium phosphate buffer (50 mM, 250 mM NaCl, pH 7.4), 3 vol% DMSO, inert atmosphere.

b Reaction conditions: 30 mM substrate, 0.5 mL whole cells containing Mb H93S at OD = 40, 22 °C, 16 h, sodium phosphate buffer (100 mM, pH 7.4), 10 vol% DMSO, inert atmosphere.

Finally, we investigated further substrates for myoglobin-catalysed ATRC. Three additional compounds were investigated in ATRC catalysed by the H93S mutant. Substrate 5, in which the benzyl substituent on the nitrogen of compound 1 was replaced with an allyl group to allow for an enhanced rotation of the amid bond, showed good conversion during catalysis with whole cells, while the performance of purified protein was lower (Table 1, entry 2). The purified protein performed the ATRC of 6, the analogue of compound 5 where methyl and bromo groups were replaced with chloro-substituents, much better than the ring closure of 5 (Table 1, entry 3). No significant differences in TTN of 6 and 7 were observed between purified protein and reaction with whole cells (Table 1, entries 3 and 4). Additionally, the formation of 6-membered ring product was catalysed, although with lower TTN compared to the 5 membered ring formation (Table 1, entry 5). Thus, the H93S mutant also performs ATRC of chlorinated compounds and 6 membered rings.

In conclusion, myoglobin was engineered by site-directed mutagenesis to catalyse ATRC, which is one of the first examples of biocatalytic ATRC and a proof that haem proteins can catalyse atom transfer of halogen atoms from and to organic compounds, thereby creating carbon-centred radicals and controlling their subsequent addition to carbon–carbon double bonds. Replacing the iron-coordinating histidine with serine, or small changes in or at the entrance to the active site, transformed the completely inactive wild-type myoglobin into an enzyme that can catalyse the addition of C–Br and C–Cl bonds across a carbon–carbon double bond. This expands the enzymatic activity of myoglobin, previously most prominently known as oxygen binding protein and as peroxidase, to atom transfer radical additions, so that the mutants might be called ATRCases (or ATRAases, as ATRC is a subtype of ATRA). Myoglobin-catalysed ATRC could be carried out with the purified protein, in crude cell lysate and in whole cell biocatalysis, whereby reactions in lysate and in whole cells yielded similar or higher turnover than with the pure protein, indicating that the protein does not have to be tediously purified when recombinantly expressed, and that the biochemical machinery of bacterial cells is beneficial for the ATRC activity. The developed mutants can serve as a starting point for directed evolution to further enhance the catalytic performance of such ATRCases. Moreover, consequent studies will focus on stereospecific radical cyclisations, taking advantage of the chiral environment that the active site of myoglobin offers. Thus, ATRCases could become powerful catalysts for the preparation of cyclic halogenated compounds, such as β-lactams, that are valuable intermediates in the synthesis of natural compounds, pharmaceuticals and other active compounds.

This work was supported by the Swiss National Science Foundation through AMBIZIONE Grants No. PZ00P2_179865 and PP00P2_172927.

## Conflicts of interest

There are no conflicts to declare.

## Supplementary Material

CC-058-D2CC03227A-s001
