# Supplementary material for: Engineered myoglobin as a catalyst for atom transfer radical cyclisation
Source: Chem Commun (Camb). 2022 Aug 31;58(78):10989–92. doi: 10.1039/d2cc03227a (PMC9521412; doi:10.1039/d2cc03227a)
Supplement: CC-058-D2CC03227A-s001 [file CC-058-D2CC03227A-s001.pdf]

Electronic Supplementary Material (ESI) for ChemComm.
This journal is © The Royal Society of Chemistry 2022

Electronic Supplementary Information

Engineered Myoglobin as a Catalyst for Atom Transfer Radical Cyclisation

Andriy Lubskyy,[a] Chao Guo,[b] Robert J. Chadwick,[b] Alke Petri-Fink,[a] Nico Bruns*[b, c] and Michela M. Pellizzoni*[a]

[a] *Adolphe Merkle Institute, University of Fribourg, Chemin des Verdiers 4,1700 Fribourg, Switzerland.*

E-mail: michela.pellizzoni@unif.ch

[b] *Department of Pure and Applied Chemistry, University of Strathclyde, 295 Cathedral Street, Glasgow G1 1XL, UK.*

[c] *Department of Chemistry, Technical University of Darmstadt, Alarich-Weiss-Straße 4, 64287 Darmstadt, Germany.*

E-mail: nico.bruns@tu-darmstadt.de

Table of Contents

Experimental section 1
- General information 1
- Experimental procedures 2
  - Genes and Cloning 2
  - Myoglobin mutant production 3
  - SDS-PAGE 8
  - Protein concentration measurement 8
  - Redox potential measurement 8
- Synthetic procedures 8
  - Biocatalytic ATRC 8
  - Biocatalytic ATRC with whole cells or cell lysate 9

Supplementary Data 18
- Supplementary Tables 18
- Supplementary Figures 19
- NMR Spectra 24

References 36

Experimental section

General information

Chemicals and solvents were purchased from commercial suppliers (Sigma Aldrich, TCI chemicals) and were used without further purification unless otherwise stated. Sterile work was performed in a Thermo Safe 2020 (ThermoFischer) laminar flow hood. The cells were grown in the shaking incubator

NB-205QMC (N-Biotek) and Orbital Shaker Incubator ES-20 (BioSan). Centrifugation was done on VWR Mega Star 1.6 R centrifuge. Optical density was measured on Ultrospec 10 Cell density meter (Biochrom). Cell lysis was done with sonicator Vibra-Cell (Sonics) with a model CV334 tip. Proteins were purified on an ÄKTA Pure fast protein chromatography system equipped with UV detector and fraction collector. UV-Vis spectra were collected on Jasco V-670 spectrophotometer. Spectra were processed and formatted using non-commercial version of Spectragryph 1.2. ^{1}H and ^{13}C NMR spectra were collected on Bruker Ultra Shield 300 MHz or Ascend 400 MHz spectrometers and referenced to residual solvent signals or TMS internal standard. GC-MS spectra were collected with an Agilent 6850 system with HP5-ms column (capillary 30.0 m x 250 µm x 0.25 µm) and Agilent MSD 5975 ESI detector. Quantification of product peaks in GC-MS was carried out by referencing the area of the GC peak of the product to the area of the internal standard and comparing this data with calibration curves obtained by injecting known amounts of each ATRC product and the internal standard. HPLC was performed with Thermo Scientific Dionex Ultimate 3000 UHPLC system with Diode array UV-Vis detector. *Escherichia coli* TOP 10, *E. coli BL21* and *E.Coli Dh5Alpha* strains used in this work were a kind gift from the research group of Prof. Thomas Ward (University of Basel). pET His6 TEV LIC cloning vector (1B) was a gift from Scott Gradia (Addgene plasmid # 29653; http://n2t.net/addgene:29653; RRID:Addgene_29653).

Experimental procedures

Genes and Cloning

The wild type (WT) sperm whale (*Physeter macrocephalus*) myoglobin gene (UniProtKB - P02185 (MYG_PHYMC) was obtained from genScript in a pUC57 vector and cloned into the empty vector pET1B (pET His6 TEV LIC cloning vector (1B)).

Myo_WT (*Physeter macrocephalus*) optimized gene sequence from GenScript:
ATGGTGCTGAGCGAGGGTGAATGGCAGCTGGTGCTGCACGTTTGGGCGAAAGTGGAGGCGGACGTTGCGGG
TCACGGCCAAGATATCCTGATTCGTCTGTTCAAAAGCCACCCGGAAACCCTGGAAAAGTTCGACCGTTTTAAAC
ACCTGAAGACCGAGGCGGAAATGAAGGCGAGCGAAGATCTGAAGAAACATGGTGTGACCGTTCTGACCGCG
CTGGGTGCGATCCTGAAGAAAAAGGGCCACCACGAGGCGGAACTGAAACCGCTGGCGCAGAGCCACGCGAC
CAAACACAAGATCCCGATTAAGTACCTGGAGTTTATTAGCGAAGCGATCATTCACGTTCTGCATAGCCGTCATC
CGGGTGACTTTGGTGCGGATGCGCAGGGTGCGATGAACAAAGCGCTGGAGCTGTTTCGTAAGGACATCGCGG
CGAAATACAAGGAACTGGGTTATCAAGGC

Myo_WT (*Physeter macrocephalus*) protein sequence:

MVLSEGEWQLVLHVWAKVEADVAGHGQDILIRLFKSHPETLEKFDRFKHLKTEAEMKASEDLKKHGVTVLTALGAI
LKKKGHHEAELKPLAQSSATKHXIPIKYLEFISEAIIHVLHSRHPGDFGADAQGAMNKALELFRKDIAAKYKELGYQG

Cloning

pET1b (Addgene) was used as the recipient plasmid for protein overexpression. In this construct, the Mb gene was fused to a polyhistidine tag following a LIC cloning protocol as described at http://qb3.berkeley.edu/qb3/macrolab/. The PCR product was used to transform TOP10 *E. coli* cells. Plasmid was isolated using EZNA DNA Miniprep kit and pET1b_Mb-WT was sent to Microsynth (Switzerland) for sequencing.

Myoglobin mutant production

PCR

Site-directed mutagenesis was performed using the Q5 Hot Start High-Fidelity DNA polymerase kit (New England BioLabs). The required DNA primers for forward and reverse strands were purchased from Microsynth. PCR reactions were performed according to the manufacturer's instructions.

Example of PCR reactions:

- 10 µL Q5 reaction buffer (X5)
- 33 µL ddH_2O
- 0.5 µL template plasmid (2 ng uL^{-1})
- 2.5 µL Forward (FW) primer (10 µM)
- 2.5 µL Backward (BW) primer (10 µM)
- 5 µL dNTPs (2.5 mM/base)
- 0.5 µL Q5 HF polymerase.

PCR Program:

- 98°C - 30 sec
- Start 30x:
    - 98 °C – 10 sec
    - 60 °C – 20 sec
    - 72 °C – 3 min
    - End cycle
    - 72 °C – 4 min
- 4 °C – hold.

The PCR mixture was purified on agarose gel, prepared by heating a 0.7% wt of agarose in 0.5x TBE Buffer(0.065 M tris at pH 7.6, 22.5 mM Boric Acid and 1.25 mM EDTA). The purified fraction (around 6kbp) was used to transform 50 µL TOP10 chemical competent cells. Cells from -80°C were thawed on ice for 30 minutes and then 1 µL of cold PCR reaction mixture was added. Cells were incubated on ice for 30 minutes, heat shocked (30 s, 42 °C), diluted with 0.9 mL of Luria-Bertani (LB) media and incubated for 1 h at 37 °C at 150 rpm. Cells were then plated on LB agar plates containing kanamycin. Bacteria were grown for 18 h at 37 °C. Individual colonies were used to inoculate 4 mL LB media with kanamycin (100 µg ml^{-1}) and grown overnight (16 h, 37°C, 180 rpm). The plasmids were purified using the EZNA DNA Miniprep kit according to the manufacturer's instructions. To confirm the mutations, the plasmids were sent for sequencing to Microsynth.

Used primers:

H93S_FW 5'-CAGAGC**AGC**GCGACCAAACACAAGATC-3'

H93S_BW 5'-GTCGC**GCT**GCTCTGCGCCAGC-3'

H93A_FW 5'-CAGAGC**GCC**GCGACCAAACACAAGATC-3'

H93A_BW 5'-GGTCGC**GGC**GCTCTGCGCCAG-3'

H93C_FW 5’-CAGAGC**TGC**GCGACCAAACACAAGATC-3’

H93C_BW 5’-GGTCGC**GCA**GCTCTGCGCCAG-3’

H93Y_FW 5’-CAGAGC**TAT**GCGACCAAACACAAGATC-3’

H93Y_BW 5’-GGTCGC**ATA**GCTCTGCGCCAG-3’

F43A_FW 5’-GAA AAG **GCC** GAC CGT TTT AAA CAC CTG-3’

F43A_BW 5’-CG GTC **GGC** CTT TTC CAG GGT TTC-3’

H64V_FW 5’-G AAG AAA **GTG** GGT GTG ACC GTT CTG ACC-3’

H64V_BW 5’-CAC ACC **CAC** TTT CTT CAG ATC TTC GCT C-3’

H64D_FW 5’-G AAG AAA **GAT** GGT GTG ACC GTT CTG ACC-3’

H64D_BW 5’-CACACC**ATC**TTTCTTCAGATCTTCGCTC-3’

Sequencing data

Mutations compared to the WT myoglobin are highlighted in bold.

>pET1B_Myo_F43A T7

CATGGGTTCTTCTCACCATCACCATCACCATGAAAACCTGTACTTCCAATCCAATGCAATGGTGCTGAGCGAGG
GTGAATGGCAGCTGGTGCTGCACGTTTGGGCGAAAGTGGAGGCGGACGTTGCGGGTCACGGCCAAGATATCC
TGATTCGTCTGTTCAAAAGCCACCCGGAAACCCTGGAAAAG**GCC**GACCGTTTTAAACACCTGAAGACCGAGGC
GGAAATGAAGGCGAGCGAAGATCTGAAGAAACATGGTGTGACCGTTCTGACCGCGCTGGGTGCGATCCTGAA
GAAAAAGGGCCACCACGAGGCGGAACTGAAACCGCTGGCGCAGAGCCACGCGACCAAACACAAGATCCCGA
TTAAGTACCTGGAGTTTATTAGCGAAGCGATCATTCACGTTCTGCATAGCCGTCATCCGGGTGACTTTGGTGCG
GATGCGCAGGGTGCGATGAACAAAGCGCTGGAGCTGTTTCGTAAGGACATCGCGGCGAAATACAAGGAACTG
GGTTATCAAGGCTAATAACATTGGAAGTGGATAACGGATCCGAATTCGAGCGCCGTCGACAAGCTTGCGGCC
GCACTCGAGCACCACCACCACCACCACTGAGATCCGGCTGCTAACAAAGCCCGAAAGGAAGCTGAGTTGGCTG
CTGCCACCGCTGAGCAATAACTAGCATAACCCCTTGGGGCCTCTAAACGGGTCTTGAGGGGTTTTTTGCTGAAA
GGAGGAACTATATCCGGATTGGCGAATGGGACGCGCCCTGTAGCGGCGCATTAAGCGCGGCGGGTGTGGTG
GTTACGCGCAGCGTGACCGCTACACTTGCCAGCGCCCTAGCGCCCGCTCCTTTCGCTTTCTTCCCTTCCTTTCTC
G

>pET1B_Myo_H93S T7
TCACCATGAAAACCTGTACTTCCAATCCAATGCAATGGTGCTGAGCGAGGGTGAATGGCAGCTGGTGCTGCAC
GTTTGGGCGAAAGTGGAGGCGGACGTTGCGGGTCACGGCCAAGATATCCTGATTCGTCTGTTCAAAAGCCAC
CCGGAAACCCTGGAAAAGTTCGACCGTTTTAAACACCTGAAGACCGAGGCGGAAATGAAGGCGAGCGAAGAT
CTGAAGAAACATGGTGTGACCGTTCTGACCGCGCTGGGTGCGATCCTGAAGAAAAAGGGCCACCACGAGGCG
GAACTGAAACCGCTGGCGCAGAGC**AGC**GCGACCAAACACAAGATCCCGATTAAGTACCTGGAGTTTATTAGC
GAAGCGATCATTCACGTTCTGCATAGCCGTCATCCGGGTGACTTTGGTGCGGATGCGCAGGGTGCGATGAACA

AAGCGCTGGAGCTGTTTCGTAAGGACATCGCGGCGAAATACAAGGAACTGGGTTATCAAGGCTAATAACATT
GGAAGTGGATAACGGATCCGAATTCGAGCGCCGTCGACAAGCTTGCGGCCGCACTCGAGCACCACCACCACC
ACCACTGAGATCCGGCTGCTAACAAAGCCCGAAAGGAAGCTGAGTTGGCTGCTGCCACCGCTGAGCAATAACT
AGCATAACCCCTTGGGGCCTCTAAACGGGTCTTGAGGGGTTTTTTGCTGAAAGGAGGAACTATATCCGGATTG
GCGAATGGGACGCGCCCTGTAGCGGCGCATTAAGCGCGGCGGGTGTGGTGGTTACGCGCAGCGTGACCGCT
ACACTTGCCAGCGCCCTAGCGCCCGCTCCTTTCGCTTTCTTCCC

>pET1B_Myo_H93C T7
TTTTGTTTAACTTTAAGAAGGAGATATACCATGGGTTCTTCTCACCATCACCATCACCATGAAAACCTGTACTTC
CAATCCAATGCAATGGTGCTGAGCGAGGGTGAATGGCAGCTGGTGCTGCACGTTTGGGCGAAAGTGGAGGC
GGACGTTGCGGGTCACGGCCAAGATATCCTGATTCGTCTGTTCAAAAGCCACCCGGAAACCCTGGAAAAGTTC
GACCGTTTTAAACACCTGAAGACCGAGGCGGAAATGAAGGCGAGCGAAGATCTGAAGAAACATGGTGTGACC
GTTCTGACCGCGCTGGGTGCGATCCTGAAGAAAAAGGGCCACCACGAGGCGGAACTGAAACCGCTGGCGCAG
AGC**TGC**GCGACCAAACACAAGATCCCGATTAAGTACCTGGAGTTTATTAGCGAAGCGATCATTCACGTTCTGC
ATAGCCGTCATCCGGGTGACTTTGGTGCGGATGCGCAGGGTGCGATGAACAAAGCGCTGGAGCTGTTTCGTA
AGGACATCGCGGCGAAATACAAGGAACTGGGTTATCAAGGCTAATAACATTGGAAGTGGATAACGGATCCGA
ATTCGAGCGCCGTCGACAAGCTTGCGGCCGCACTCGAGCACCACCACCACCACCACTGAGATCCGGCTGCTAA
CAAAGCCCGAAAGGAAGCTGAGTTGGCTGCTGCCACCGCTGAGCAATAACTAGCATAACCCCTTGGGGCCTCT
AAACGGGTCTTGAGGGGTTTTTTGCTGAAAGGAGGAACTATATCCGGATTGGCGAATGGGACGCGCCCTGTA
GCGGCGCATTAAGCGCGGCGGGTGTGGTGGTTACGCGCAGCGTGACCGCTACACTTGCCAGCGCCCTAGCGC
CCGCTCCTTTCGCTTTCTTCCCTTCCTTTCTCGCCACGTTCGCCGGCTTTCCCCGTCAAGCTCTAANNGGGGGCT
CCCTTTAGGGTTCCGATTT

>pET1B_Myo_F43A_H93S T7
TCCCTCTAGAATAATTTTGTTTAACTTTAAGAAGGAGATATACCATGGGTTCTTCTCACCATCACCATCACCATG
AAAACCTGTACTTCCAATCCAATGCAATGGTGCTGAGCGAGGGTGAATGGCAGCTGGTGCTGCACGTTTGGGC
GAAAGTGGAGGCGGACGTTGCGGGTCACGGCCAAGATATCCTGATTCGTCTGTTCAAAAGCCACCCGGAAAC
CCTGGAAAAG**GCC**GACCGTTTTAAACACCTGAAGACCGAGGCGGAAATGAAGGCGAGCGAAGATCTGAAGA
AACATGGTGTGACCGTTCTGACCGCGCTGGGTGCGATCCTGAAGAAAAAGGGCCACCACGAGGCGGAACTGA
AACCGCTGGCGCAGAGC**AGC**GCGACCAAACACAAGATCCCGATTAAGTACCTGGAGTTTATTAGCGAAGCGA
TCATTCACGTTCTGCATAGCCGTCATCCGGGTGACTTTGGTGCGGATGCGCAGGGTGCGATGAACAAAGCGCT
GGAGCTGTTTCGTAAGGACATCGCGGCGAAATACAAGGAACTGGGTTATCAAGGCTAATAACATTGGAAGTG
GATAACGGATCCGAATTCGAGCGCCGTCGACAAGCTTGCGGCCGCACTCGAGCACCACCACCACCACCACTGA
GATCCGGCTGCTAACAAAGCCCGAAAGGAAGCTGAATTGGCTGCTGCCACCGCTGAACAATAACTAGCATAAC
CCCTTGGGGSCTCTAAACGGGTCTTGARGGGTTTTTTGCTGAAAGGAGGAACTATATCCGGATTGGCGAATGG
GACGCGCCCTGTAGCGGCGCATTAAGCGCGGCGGGTGTGGTGGTTACGCGCAGCGTGACCGCTACACTTGCC
AGCGCCCTAGCGCCCGCTCCTTTCGCTTTCTTCCCTTCCTTTCTCGCCACGTTCGCCGGCTTTCCCCGTCAAGCTC
TAAATCGGGGGCTCCCTTTAGGGTTCCGATTTAGTGCTTTACGGCACCTCGACCCCAAAAAACTTGATTAGGGT
GATGGTTCACGTAGTGGGCCATCGCCCTGATAGACGGTTTTTCGCCCTTTGACGTTGGAGTCCACGTTCTTTAA
TAGTGGACTCTTGTTCCAACTGGAACAACMCTCAACCC

>pET1B_Myo_H93A T7
TCCCTCTAGAATAATTTTGTTTAACTTTAAGAAGGAGATATACCATGGGTTCTTCTCACCATCACCATCACCATG
AAAACCTGTACTTCCAATCCAATGCAATGGTGCTGAGCGAGGGTGAATGGCAGCTGGTGCTGCACGTTTGGGC
GAAAGTGGAGGCGGACGTTGCGGGTCACGGCCAAGATATCCTGATTCGTCTGTTCAAAAGCCACCCGGAAAC
CCTGGAAAAGTTCGACCGTTTTAAACACCTGAAGACCGAGGCGGAAATGAAGGCGAGCGAAGATCTGAAGAA

ACATGGTGTGACCGTTCTGACCGCGCTGGGTGCGATCCTGAAGAAAAAGGGCCACCACGAGGCGGAACTGAA
ACCGCTGGCGCAGAGC**GCC**GCGACCAAACACAAGATCCCGATTAAGTACCTGGAGTTTATTAGCGAAGCGATC
ATTCACGTTCTGCATAGCCGTCATCCGGGTGACTTTGGTGCGGATGCGCAGGGTGCGATGAACAAAGCGCTGG
AGCTGTTTCGTAAGGACATCGCGGCGAAATACAAGGAACTGGGTTATCAAGGCTAATAACATTGGAAGTGGA
TAACGGATCCGAATTCGAGCGCCGTCGACAAGCTTGCGGCCGCACTCGAGCACCACCACCACCACCACTGAGA
TCCGGCTGCTAACAAAGCCCGAAAGGAAGCTGARTTGGCTGCTGCCACCGCTGAGCAATAACTAGCATAACCC
CTTGGGGCCTCTAAACGGGTCTTGARGGGTTTTTTGCTGAAAGGAGGAACTATATCCGGATTGGCGAATGGGA
CGCGCCCTGKAGCGGCGCATTAAGCGCGGCGGGTGTGGTGGTTACGCGCAGCGTGACCGCTACACTTGCCAG
CGCCCTAGCGCCCGCTCCTTTCGCTTTCTTCCCTTCCTTTCTCGCCACGTTCGCCGGCTTTCCCCGTCAAGCTCTA
AATCGGGGGCTCCCTTTAGGGTTCCGATTTAGTGCTTTACGGCACCTCGACCCCAAAAAACTTGATTAGGGTGAT
GGTTCACGTAGTGGGCCATCGCCCTGAWAGACGGTTTTTCGCCCTTTGACGTTGGAGTCNNGTTCTTTATAAT
GGACTCTTGTTCCAACTGGAACAMMCTCAACCC

>pET1B_Myo_H93Y T7
NNNNTCCCTCTAGAATAATTTTGTTTAACTTTAAGAAGGAGATATACCATGGGTTCTTCTCACCATCACCATCAC
CATGAAAACCTGTACTTCCAATCCAATGCAATGGTGCTGAGCGAGGGTGAATGGCAGCTGGTGCTGCACGTTT
GGGCGAAAGTGGAGGCGGACGTTGCGGGTCACGGCCAAGATATCCTGATTCGTCTGTTCAAAAGCCACCCGG
AAACCCTGGAAAAGTTCGACCGTTTTAAACACCTGAAGACCGAGGCGGAAATGAAGGCGAGCGAAGATCTGA
AGAAACATGGTGTGACCGTTCTGACCGCGCTGGGTGCGATCCTGAAGAAAAAGGGCCACCACGAGGCGGAAC
TGAAACCGCTGGCGCAGAGC**TAT**GCGACCAAACACAAGATCCCGATTAAGTACCTGGAGTTTATTAGCGAAGC
GATCATTCACGTTCTGCATAGCCGTCATCCGGGTGACTTTGGTGCGGATGCGCAGGGTGCGATGAACAAAGCG
CTGGAGCTGTTTCGTAAGGACATCGCGGCGAAATACAAGGAACTGGGTTATCAAGGCTAATAACATTGGAAG
TGGATAACGGATCCGAATTCGAGCGCCGTCGACAAGCTTGCGGCCGCACTCGAGCACCACCACCACCACCACT
GAGATCCGGCTGCTAACAAAGCCCGAAAGGAAGCTGAGTTGGCTGCTGCCACCGCTGAGCAATAACTAGCAT
AACCCCTTGGGGCCTCTAAACGGGTCTTGAGGGGTTTTTTGCTGAAAGGAGGAACTATATCCGGATTGGCGAA
TGGGACGCGCCCTGTAGCGGCGCATTAAGCGCGGCGGGTGTGGTGGTTACGCGCAGCGTGACCGCTACACTT
GCCAGCGCCCTAGCGCCCGCTCCTTTCGCTTTCTTCCCTTCCTTTCTCGCCACGTTCGCCGGCTTTCCCCGTCAAG
CTCTAAATCGGGGGCTCCCTTTAGGGTTCCGATTTAGTGCTTTACGGCACCTCGACCCCAAAAAACTTGATTAG
GGTGATGGTTCACGTAGTGGGCCATCGCCCTGATAGACGGTTTTTCGCCCTTTGACGTTGGAGTCCACGTTCTT
TATAGTGGACTCTTGTTCCAACTGGAACACMCTCAACCTATCTCGGNCTATTCTTTGATTATAAGGGATTTGCC
GATTTCGGCCTWTGGTTAAAAATGACTGATTAACAAAATTTACSSGATTTTACAACTAGWACGTTACATTTCAG
GGGCMTTTTCGGGAAAT

>pET1B_Myo_H64V T7
CTCTAGAATAATTTTGTTTAACTTTAAGAAGGAGATATACCATGGGTTCTTCTCACCATCACCATCACCATGAAA
ACCTGTACTTCCAATCCAATGCAATGGTGCTGAGCGAGGGTGAATGGCAGCTGGTGCTGCACGTTTGGGCGAA
AGTGGAGGCGGACGTTGCGGGTCACGGCCAAGATATCCTGATTCGTCTGTTCAAAAGCCACCCGGAAACCCTG
GAAAAGTTCGACCGTTTTAAACACCTGAAGACCGAGGCGGAAATGAAGGCGAGCGAAGATCTGAAGAAA**GT**
GGGTGTGACCGTTCTGACCGCGCTGGGTGCGATCCTGAAGAAAAAGGGCCACCACGAGGCGGAACTGAAACC
GCTGGCGCAGAGCCACGCGACCAAACACAAGATCCCGATTAAGTACCTGGAGTTTATTAGCGAAGCGATCATT
CACGTTCTGCATAGCCGTCATCCGGGTGACTTTGGTGCGGATGCGCAGGGTGCGATGAACAAAGCGCTGGAG
CTGTTTCGTAAGGACATCGCGGCGAAATACAAGGAACTGGGTTATCAAGGCTAATAACATTGGAAGTGGATAA
CGGATCCGAATTCGAGCGCCGTCGACAAGCTTGCGGCCGCACTCGAGCACCACCACCACCACCACTGAGATCC
GGCTGCTAACAAAGCCCGAAAGGAAGCTGAGTTGGCTGCTGCCACCGCTGAGCAATAACTAGCATAACCCCTT
GGGGCCTCTAAACGGGTCTTGAGGGGTTTTTTGCTGAAAGGAGGAACTATATCCGGATTGGCGAATGGGACG
CGCCCTGTAGCGGCGCATTAAGCGCGGCGGGTGTGGTGGTTACGCGCAGCGTGACCGCTACACTTGCCAGCG
CCCTAGCGCCCGCTCCTTTCGCTTTCTTCCCTTCCTTTCTCGCCACGTTCGCCGGCTTTCCCCGTCAAGCTCTAAA
TCGGGGGCTCCCTTTAGGGTTCCGATTTAGTGCTTTACGGCACCTCGACCCCAAAAAACTTGATTAGGGTGATG

GTTCACGTAGTGGGCCATCGCCCTGATAGACGGTTTTTCGCCCTTTGACGTTGGAGTCNCGTTCTTTATAATGG
ACTTCTGTTCCAAACTGGAACAAMCTCAACCTATCTCGGCTATTCTTTGATTAAAAGGGATTTGCCGAATTCGG
CCTATGGGTTAAAAA

>pET1B_Myo_H64V_H93S T7
ATTTTGTTTACTTTAAGAAGGAGATATACCATGGGTTCTTCTCACCATCACCATCACCATGAAAACCTGTACTTC
CAATCCAATGCAATGGTGCTGAGCGAGGGTGAATGGCAGCTGGTGCTGCACGTTTGGGCGAAAGTGGAGGC
GGACGTTGCGGGTCACGGCCAAGATATCCTGATTCGTCTGTTCAAAAGCCACCCGGAAACCCTGGAAAAGTTC
GACCGTTTTAAACACCTGAAGACCGAGGCGGAAATGAAGGCGAGCGAAGATCTGAAGAAA**GTG**GGTGTGAC
CGTTCTGACCGCGCTGGGTGCGATCCTGAAGAAAAAGGGCCACCACGAGGCGGAACTGAAACCGCTGGCGCA
GAGC**AGC**GCGACCAAACACAAGATCCCGATTAAGTACCTGGAGTTTATTAGCGAAGCGATCATTCACGTTCTG
CATAGCCGTCATCCGGGTGACTTTGGTGCGGATGCGCAGGGTGCGATGAACAAAGCGCTGGAGCTGTTTCGT
AAGGACATCGCGGCGAAATACAAGGAACTGGGTTATCAAGGCTAATAACATTGGAAGTGGATAACGGATCCG
AATTCGAGCGCCGTCGACAAGCTTGCGGCCGCACTCGAGCACCACCACCACCACCACTGAGATCCGGCTGCTA
ACAAAGCCCGAAAGGAAGCTGAGTTGGCTGCTGCCACCGCTGAGCAATAACTAGCATAACCCCTTGGGGCCTC
TAAACGGGTCTTGAGGGGTTTTTTGCTGAAAGGAGGAACTATATCCGGATTGGCGAATGGGACGCGCCCTGT
AGCGGCGCATTAAGCGCGGCGGGTGTGGTGGTTACGCGCAGCGTGACCGCTACACTTGCCAGCGCCCTAGCG
CCCGCTCCTTTCGCTTTCTTCCCTTCCTTTCTCGCCACGTTCGCCGGCTTTCCCCGTCAAGCTCTAAATCGGGGGC
TCCCTTTAGGGTTCCGATTTAGTGCTTTACGGCACCTCGACCCCAAAAAACTTGATTAGGGTGATGGTTCACGT
AGTGGGCCATCGCCCTGATAGACGGTTTTTCGCCCTTTGACGTTGGAGTCCACGTTCTTTAATAGTGGACTCTTG
TTCCAAACTGGAACAACACTCAACCCTATCTCGGTCTATTCTTTTGATTTAAAGGGATTTTGCCGATTTCGGCCT
ATGGTTAAAAAATGAGCTGATTT

Protein expression and purification

For expression of WT Mb and of Mb mutants, pET1b_Mb plasmid were used to transform BL21 (DE3) *E. coli* competent cells. Transformed cells were plated onto an LB agar plate containing 100 µg mL^{-1} kanamycin. A single colony of freshly transformed cells was cultured overnight in 5 mL of LB medium containing 100 µg mL^{-1} kanamycin. 4 mL of the culture was used to inoculate a flask of Rich Media (250 mL LB, 12.5 mL 20X M9 salt solution (150.4 g L^{-1} Na_2HPO_4*$2H_2O$, 60 g L^{-1} KH_2PO_4, 10 g L^{-1} NaCl, 10 g L^{-1} NH_4Cl), 5 mL 20 wt% glucose, and 250 µl of kanamycin (100 µg/mL)). The culture was incubated for ~4 h at 37 °C with shaking at 180 rpm. When the OD_{600} of the culture reached 1-1.5, isopropyl β-D-thiogalactoside (IPTG) and δ-aminolevulinic acid were added to a final concentration of 1 mM and 0.3 mM, respectively. The culture was incubated for an additional 22 h at 27 °C with shaking at 180 rpm. Then, the bacterial cells were pelleted by centrifugation in 250 ml flasks at 4 °C and 4500 g. The cell pellets were suspended in 20 mL NTA buffer (50 mM sodium phosphate, 250 mM NaCl, pH 8.0). Cells were lysed by sonication at 60% power (4 cycles of 30 s pulse and 150 s break). Cell lysates were centrifuged at 4000 g for 20 min, and the supernatants were loaded on 5 ml HisTrap FF Crude column (Cytiva). His-tagged Mb variants were eluted with 50 mM sodium phosphate buffer (250 mM NaCl, pH 7.4) containing 300 mM imidazole on an ÄKTA Pure fast protein chromatography system. The fractions were monitored at 214, 280, and 400 nm, and collected using a BioFrac fraction collector. Protein-containing fractions were analysed by sodium dodecylsulfate polyacrylamide gel electrophoresis (SDS-PAGE). The buffer was exchanged against sodium phosphate buffer (50 mM, pH 7.4) or PBS-Br buffer (10 mM phosphate, 100 mM NaBr) by concentrating the protein solution with Amicon Ultra-15 (10 kDa cut off) centrifugal filter devices (Merck Millipore) several times or dialysed twice against 10 L of the buffer with Spectra/Por® Dialysis Membrane (MWCO: 6-8 kDa).

SDS-PAGE

Running gel (12%) and stacking gel were prepared using following recipe:

Reagent	Running gel	Stacking gel
**H_2O**	5 mL	3.4 mL
**Acrylamide/bisacrylamide (30% 37.5:1; Bio-Rad)**	6 mL	1 mL
**Tris–HCl (1.5 M, pH 8.8, running gel) (0.5 M, pH 6.8, stacking gel)**	3.7 mL	1.5 mL
**SDS, 20%**	75 µL	30 µL
**N,N,N′,N′-tetramethylethylene diamine (TEMED)**	6 µL	6 µL
**Ammonium persulfate (APS), 15%**	40 µL	40 µL

Protein samples were mixed with LPS loading buffer and loaded into gel lanes starting with protein ladder solution. The SDS-PAGE experiment was performed in SDS-PAGE running buffer (25 mM Tris, 192 mM glycine and 0.1% SDS, pH 8.3) at 120 V for at least 90 minutes. Gels were stained with standard Coomassie stain protocol.

Protein concentration measurement

Protein concentration was determined with a pyridine hemochromagen assay.[1] The protein solution (20µL) was diluted 5-times with an aqueous solution of 0.2 M NaOH, 50 vol% pyridine and 0.01 M potassium ferricyanide (80µL). UV-vis spectra were recorded in the range of 500-600 nm. The solution was reduced with 1 µL of a 10 mM solution of sodium dithionate (NaDT), and the spectral scans were taken every 30 seconds for 5 min. The concentration was determined based on the absorbance differences at 557 nm of reduced and at 540 nm of oxidised sample peaks using reported equations.

Redox potential measurement

Redox potentials were determined as reported by Efimov et al.[2] 20 µL of 1 M glucose solution, 10 µL of 0.3 mM xanthine solution, 10 µL of 1 mg ml^{-1} catalase from bovine liver (>10000 units mg^{-1} protein, Sigma Aldrich) were added to 0.8 mL buffer (50 mM sodium phosphate, 250 mM NaCl, pH 7.4) in a sealable 1 cm quartz semi-micro cuvette equipped with a septum (Hellma). The mixture was degassed for 5 min by bubbling argon through the cuvette by means of syringe needles. Solutions of a myoglobin variant (typically 30mM) and dye (typically 1 mg ml^{-1} of methylene blue, nile blue or phenosaphranine, Figure SI1) were then added to this solution, with the concentrations adjusted by titration to give a peak absorbance of around 0.1-0.5. Glucose oxidase from *Aspergillus niger* (135200 units g^{-1} solid; Sigma-Aldrich) and 10 µL of xanthine oxidase(50 mg ml^{-1}) from bovine milk (0.08 units mg^{-1} solid ; Sigma-Aldrich) solution were added. The headspace was purged with argon for a few seconds and UV-Vis spectra were recorded in the range from 350 to 740 nm every 90 s for 1 h or until full reduction of the protein. Few specs of NaDT crystals were later added to fully reduce the dye and protein. The UV-vis spectrum was recorded once more. Redox potentials were calculated from the data (Figure SI 1) as described by Efimov et al.[2]

Synthetic procedures

Biocatalytic ATRC

The solution of myoglobin mutant (200 µL, 30 µM) in PBS-Br buffer (10 mM phosphate, 100 mM NaBr, pH 7.4) or phosphate buffer(50 mM phosphate, 250 mM NaCl, pH 7.4) was degassed with

argon in a closed 2 mL screwcap vial with a septum containing a small magnetic stir bar. A degassed solution of sodium ascorbate (100 µL, 300 mM) was added via a syringe. 10 µL of a 1.2M stock solution of substrate in DMSO (that resulted in 3 vol% DMSO solution) were added and the vial was sealed with parafilm and stirred on a stir plate with a water bath at 40°C for 16 h with stirring at 120 rpm. A solution (50 µL) of 0.1 M benzodioxole in methanol was added as internal standard. The resulting mixture was extracted with 1 mL of ethyl acetate (EtOAc). The organic phase was dried over $MgSO_4$. 500 µL of the sample was diluted with 500 µL EtOAc. The extract was injected into a GC/MS (parameters described below during compound characterisation). The total turnover number (TTN) were calculated using calibration curves obtained by injecting known amounts of each ATRC product and referencing the area of the GC peak of the product to the area of the internal standard.

Biocatalytic ATRC with whole cells or cell lysate

Myoglobin variants were recombinantly expressed in BL21 (DE3) *E. coli* competent cells as described above. After centrifugation, the cell pellets were resuspended in sodium phosphate buffer (50 mM, pH 7.4) to OD_{600} = 40, and lysed using sonication in case of experiments with lysed cells. A suspension (400 µL) of these myoglobin expressing cells (or cell lysate) was added to a 2 ml vial on ice. 50 µL of a solution of reducing agent (300 mM sodium ascorbate or 300 mM glucose) in water, as well as 50 µL of a 0.24 mM substrate solution in DMSO were added(as a result 10 vol% DMSO solution was created). The vial was closed with a screw cap with a septum, the mixture was degassed by bubbling with argon for 1 min, sealed with parafilm and shaken overnight (16 h) at 1000 RPM at 25°C. After addition of internal standard (50 µL of 0.1 M benzodioxole in methanol), the solution was extracted once with 1 ml EtOAc. The organic phase was dried with $MgSO_4$ and 0.5 ml of it was diluted with 0.5 ml of EtOAc. The extract was injected into a GC/MS (details see above) and the TTN was calculated using calibration curves as detailed above.

N-allyl-N-benzyl-2-bromo-2-methylpropanamide (1)

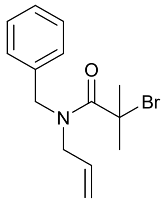

N-allyl-N-benzyl-2-bromo-2-methylpropanamide

Triethylamine (759 mg, 1.05 ml, mmol, 7.5 eq.) was added to a solution of N-benzylallylamine (736 mg, 0.783 ml, 1 eq., 5 mmol) in DCM (30 ml) at 0 °C. After 20 min, alpha-bromoacetyl bromide (1150 mg, 0.618 ml, 1 eq., 1.5 mmol) was added dropwise through a dropping funnel. The reaction mixture was allowed to warm to room temperature. After 4 h, the reaction was quenched with saturated NH_4Cl (2x50 ml) and partitioned between saturated $NaHCO_3$ solution and DCM. The organic layer was collected, and the aqueous layer was extracted using DCM (2 × 50 mL). The combined organic extracts were dried over $MgSO_4$, filtered, and concentrated *in vacuo* to yield the product as a mixture of rotamers. The compound was purified by flash chromatography on silica gel with a mixture of hexane/EtOAc (85:15) as eluent. The solvent was removed *in vacuo* and the compound was characterised by NMR spectroscopy and compared with data reported before.[3]

Yield 0.72 g (84%), colourless oil

^1H NMR ($CDCl_3$, 400 MHz, 363 K): 7.16−7.06 (5H, m), 5.69−5.61 (1H, m) 5.01 (1H, dd, J 10.3, 1.4 Hz), 4.98 (1H, dd, J 17.2, 1.4 Hz), 4.67 (2H, s), 4.02 (2H, d, J 5.5 Hz), 1.86 (6H, s).

^{13}C NMR (CDCl3, 101 MHz): δ 170.3, 137.6, 133.8, 128.9, 128.0, 127.5, 117.4, 57.9, 50.7, 50.4, 33.2.

N,N-diallyl-2,2,2-trichloroacetamide (2)

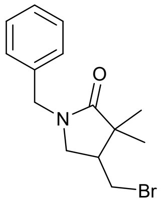

1-benzyl-4-(bromomethyl)-3,3-dimethylpyrrolidin-2-one

To the solution of 1-benzyl-4-(bromomethyl)-3,3-dimethylpyrrolidin-2-one (107 mg, 0.36 mmol) in MeOH (2.6 mL), Cu(TPMA)SO_4 (1 mol %, 3.6×10^{-3} mmol, 0.36 mL of 0.01 M stock solution in MeOH) was added, followed by $NaBH_4$ (~15 mol %, 2 mg). The reaction was monitored by TLC. After expiration of starting material, the crude solution was filtered through a silica plug and concentrated *in vacuo* to give the crude product. It was purified on a flash silica column with mixture of hexane/EtOAc (85:15) as eluent. The solvent was removed *in vacuo* and the compound was characterised by NMR spectroscopy and compared with data reported before.[3] A calibration curve for GC was measured with benzodioxol as internal standard.

Yield 76 mg (72%), colourless oil

^{1}H NMR ($CDCl_3$, 300 MHz): δ 7.32−7.15 (5H, m, Ar), 4.52 (1H, d, J 14.4 Hz), 4.35 (1H, d, J 14.4 Hz), 3.46 (1H, dd, J 10.0, 4.8 Hz), 3.35 (1H, dd, J 10.0, 7.5 Hz), 3.22 (1H, t, J 10.5 Hz), 2.88 (1H, t, J 10.2 Hz), 2.40 (1H, m), 1.24 (3H, s), 0.99 (3H, s).

^{13}C NMR ($CDCl_3$, 101 MHz): δ 178.44, 136.34, 128.78, 128.07, 127.68, 48.90, 46.70, 46.10, 44.04, 31.41, 24.27, 18.36.

GC calibration curve of substate 2:

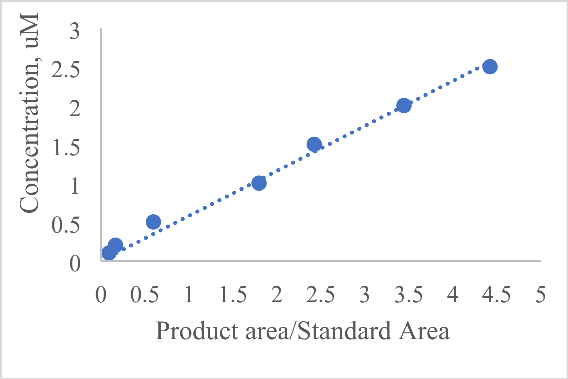

N-benzyl-2-bromo-N-(3-cyclopropylallyl)-2-methylpropanamide (3)

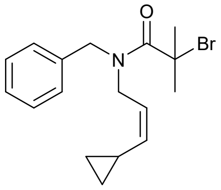

(*Z*)-*N*-benzyl-2-bromo-*N*-(3-cyclopropylallyl)-2-methylpropanamide

Triethylamine (0.313 ml, 2.5 mmol, 1.5 eq,) was added to a solution of N-benzyl-3-cyclopropylprop-2-en-1-amine (281 mg, 1.5 mmol, 1 eq) in DCM (15 ml) at 0 °C. After 20 min, α-bromoisobuturyl

bromide (0.185 ml, 1.5 mmol, 1 eq) was added dropwise through a dropping funnel. The reaction mixture was allowed to warm to room temperature. After 4 h, the reaction was quenched with saturated NH_4Cl and partitioned between saturated $NaHCO_3$ and DCM. The organic layer was collected, and the aqueous layer was extracted using DCM (2 × 50 mL). The combined organic extracts were dried over $MgSO_4$, filtered, and concentrated *in vacuo* to yield product as a single cis isomer. The compound was purified by flash chromatography on silica gel with a mixture of Hexane/EtOAc (85:15) as eluent. The solvent was removed *in vacuo* and the compound was characterised by NMR spectroscopy and compared with data reported before.[6]

Yield 370 mg (73%), colorless oil

^{1}H NMR (400 MHz, Chloroform-*d*) : δ 7.40 – 7.14 (m, 5H), 5.59 – 5.38 (m, 2H), 4.59 – 4.34 (m, 2H), 3.42 – 3.27 (m, 2H), 3.18 (dd, *J* = 9.9, 7.7 Hz, 1H), 3.02 – 2.89 (m, 1H), 1.17 (s, 3H), 0.97 (s, 3H).

^{13}C NMR (101 MHz, Chloroform-*d*) δ 170.46, 138.78, 137.12, 128.62, 127.19, 121.66, 57.25, 49.99, 48.51, 32.95, 13.44, 6.80.

1-benzyl-4-(4-bromobut-1-en-1-yl)-3,3-dimethylpyrrolidin-2-one (4)

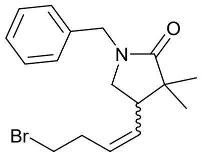

1-benzyl-4-(4-bromobut-1-en-1-yl)-3,3-dimethylpyrrolidin-2-one

To a solution of N-benzyl-2-bromo-N-(3-cyclopropylallyl)-2-methylpropanamide (101 mg, 0.3 mmol, 1 eq) in MeOH (2.6 mL), Cu(TPMA)SO_4 (1 mol %, 3.6 × 10^{-3} mmol, 0.36 mL of 0.01 M stock solution) was added followed by $NaBH_4$ (~15 mol %, 2 mg). The reaction was monitored with TLC. After expiration of starting material peaks, the crude solution was filtered through a silica plug and concentrated *in vacuo* to give the crude mixture. The product was purified on a flash silica column with a mixture of hexane and EtOAc (80:20) as eluent. The solvent was removed *in vacuo* and the compound was characterised by NMR spectroscopy and compared with data reported before.[6] A calibration curve for GC was measured with benzodioxol as internal standard.

Yield 53 mg (52%), yellow oil

1H NMR (400 MHz, Chloroform-*d*): δ 7.31 (d, J = 4.4 Hz, 5H), 7.25 – 7.19 (m, 1H), 5.63 (dt, J = 15.3, 6.4 Hz, 1H), 5.15 (ddt, J = 15.3, 8.7, 1.4 Hz, 1H), 3.77 (s, 2H), 3.20 (dd, J = 6.4, 1.4 Hz, 2H), 0.73 – 0.60 (m, 2H), 0.34 (dt, J = 6.4, 4.4 Hz, 2H).

^{13}C NMR (101 MHz, Chloroform-*d*) δ 179.13, 136.66, 130.31, 129.73, 128.70, 128.07, 127.55, 48.28, 47.63, 46.59, 44.10, 35.73, 32.38, 23.41, 19.16.

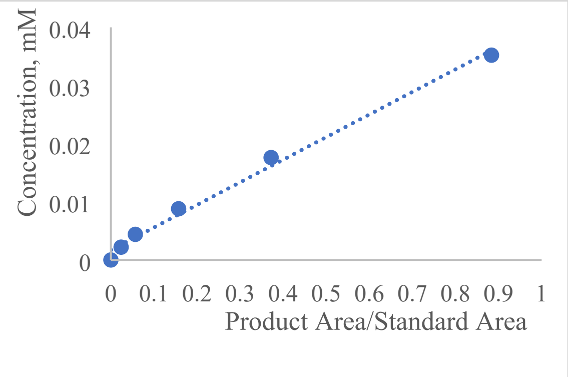

N,N-diallyl-2-bromo-2-methylpropanamide (5)

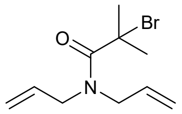

N,N-diallyl-2-bromo-2-methylpropanamide

Triethylamine (0.313 ml, 1.5 eq, 2.5 mmol) was added to a solution of diallylamine (146 mg, 1 eq, 1.5 mmol) in DCM (15ml) at 0 °C. After 20 min, α-bromoisobuturyl bromide (345mg, 0.185 ml, 1 eq, 1.5 mmol) was added dropwise through a dropping funnel. The reaction mixture was allowed to warm to room temperature. After 4 h, the reaction was quenched with saturated NH_4Cl and partitioned between saturated $NaHCO_3$ and DCM. The organic layer was collected, and the aqueous layer was extracted using DCM (2 × 50 mL). The combined organic extracts were dried over $MgSO_4$, filtered, and concentrated *in vacuo* to yield the product. The compound was purified by flash chromatography on silica gel with a mixture of hexane/EtOAc (75:25) as eluent. The solvent was removed *in vacuo* and the compound was characterised by NMR spectroscopy and elemental analysis.

Yield 254 mg (69%), colourless oil

^{1}H NMR (400 MHz, Chloroform-*d*) δ 6.00 – 5.71 (m, 1H), 5.20 (d, *J* = 17.5 Hz, 1H), 4.37 (m, 1H), 3.99 (m, 1H), 1.99 (s, 3H).

^{13}C NMR (101 MHz, Chloroform-*d*): δ 170.18, 132.90, 117.04, 57.15, 32.82.

Elemental Analysis (Predicted): C, 48.80; H, 6.55; N, 5.69, Elemental Analysis (Acquired): C, 48.9; H, 6.56; N, 5.36.

1-allyl-4-(bromomethyl)-3,3-dimethylpyrrolidin-2-one (5`)

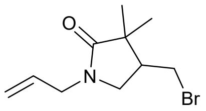

1-allyl-4-(bromomethyl)-3,3-dimethylpyrrolidin-2-one

To a solution of 1-allyl-4-(bromomethyl)-3,3-dimethylpyrrolidin-2-one (0.36 mmol) in MeOH (2.6 mL), Cu(TPMA)SO_4 (1 mol %, 3.6 × 10^{-3} mmol, 0.36 mL of 0.01 M stock solution in MeOH) was added, followed by $NaBH_4$ (~15 mol %, 2 mg). The reaction was monitored with TLC. After expiration of starting material peaks, the crude solution was filtered through a silica plug and concentrated *in vacuo* to give the crude mixture. The product was purified on a flash silica column with mixture of hexane and EtOAc (50:50) as eluent, and was characterised by NMR spectroscopy. A calibration curve for GC was measured with benzodioxol as internal standard.

Yield 34 mg (46%), colourless oil

^{1}H NMR (400 MHz, Chloroform-*d*) δ 5.72 (ddt, *J* = 16.6, 10.4, 6.2 Hz, 1H), 5.26 – 5.08 (m, 2H), 3.90 (dt, *J* = 6.1, 1.4 Hz, 2H), 3.56 – 3.42 (m, 2H), 3.31 (dd, *J* = 10.8, 10.1 Hz, 1H), 3.03 (dd, *J* = 10.1, 8.6 Hz, 1H), 2.55 – 2.34 (m, 1H), 1.62 (s, 1H), 1.23 (s, 3H), 1.01 (s, 3H).

^{13}C NMR (101 MHz, Chloroform-*d*) δ 178.20, 132.20, 118.24, 49.02, 46.17, 45.30, 44.13, 31.51, 24.31, 18.44.

GC calibration curve of substate 5`:

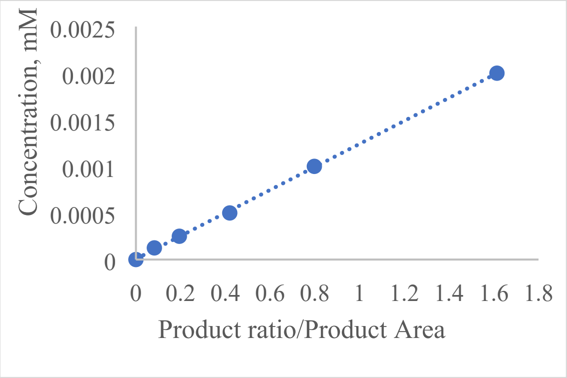

N,N-diallyl-2,2,2-trichloroacetamide (6)

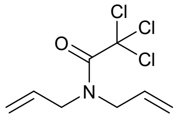

N,N-diallyl-2,2,2-trichloroacetamide

Triethylamine (1518 mg, 2.08 ml, 7.5 mmol, 7.5 eq) was added to a solution of diallylamine (486 mg, 0.616 ml, 1eq, 5 mmol) in DCM (30 ml) at 0 °C. After 20 min, trichloroacetyl chloride (1364 mg, 0.842 ml, 1 eq, 1.5 mmol) was added dropwise through a dropping funnel. The reaction mixture was allowed to warm to room temperature. After 4 h, the reaction was quenched with saturated NH_4Cl and partitioned between saturated $NaHCO_3$ and DCM. The organic layer was collected, and the aqueous layer was extracted using DCM (2 × 50 mL). The combined organic extracts were dried over $MgSO_4$, filtered, and concentrated *in vacuo* to yield product. The compound was purified by flash chromatography on silica gel with a mixture of hexane/EtOAc (75:25) as eluent. The solvent was removed *in vacuo* and the compound was characterised by NMR spectroscopy and compared with data reported before.[4]

Yield 938 g (77%), light-yellow oil

^{1}H NMR (400 MHz, Chloroform-*d*) δ 5.97 – 5.64 (m, 2H), 5.42 – 5.08 (m, 4H), 4.35 (s, 2H), 4.04 (s, 2H).

^{13}C NMR (101 MHz, Chloroform-*d*) δ 160.30, 132.08, 131.09, 119.35, 118.15, 93.04, 51.65, 49.81.

1-allyl-3,3-dichloro-4-(chloromethyl)pyrrolidin-2-one (6`)

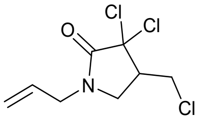

1-allyl-3,3-dichloro-4-(chloromethyl)pyrrolidin-2-one

The reaction was performed under inert atmosphere with oven dried glassware. α-halo-amide substrate (0.2 mmol, 48.5 mg, 1 eq), CuCl (6 mg, 0.06 mmol, 0.3 eq), TPMA (17.4 mg, 0.06 mmol, 0.3 eq) and a magnetic stir bar was added to a solution of Cu(I) chloride and TPMA. The reaction was sealed, purged with argon, then 3 ml of anhydrous toluene were added. The mixture was stirred under heating in an oil bath (80 °C). After reaction completion according to TLC, the solution was filtered through a silica plug, and concentrated *in vacuo* to give the crude product which was purified on a flash silica column with mixture of hexane and EtOAc (60:40) as eluent and was characterised by NMR spectroscopy and compared with data reported before.[4] A calibration curve for GC was measured with benzodioxol as internal standard.

Yield 34 mg (70%), colourless oil

^{1}H NMR (400 MHz, Chloroform-*d*) δ 5.74 (ddt, *J* = 17.0, 10.2, 6.2 Hz, 1H), 5.36 – 5.19 (m, 2H), 4.11 – 3.87 (m, 3H), 3.74 (dd, *J* = 11.2, 10.1 Hz, 1H), 3.58 (dd, *J* = 10.2, 6.9 Hz, 1H), 3.22 (dd, *J* = 10.2, 8.2 Hz, 1H)

^{13}C NMR (101 MHz, Chloroform-*d*) δ 165.75, 130.47, 119.74, 83.63, 51.72, 47.42, 46.44, 41.09.

GC calibration curve of substate 6`:

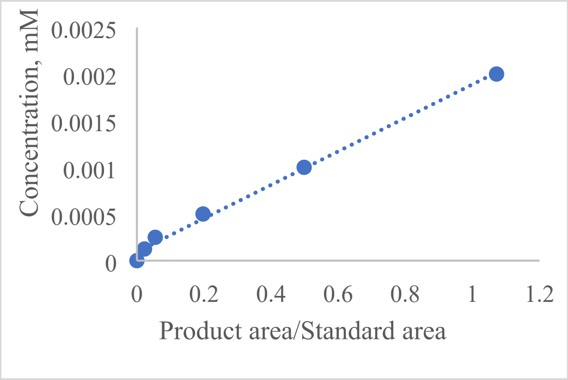

N-allyl-N-benzyl-2,2,2-trichloroacetamide (7)

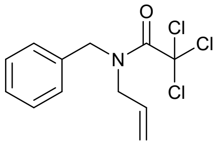

N-allyl-N-benzyl-2,2,2-trichloroacetamide

Triethylamine (304 mg, 0.418 ml, 3 mmol, 1.5 eq) was added to a solution of N-benzylallylamine (294 mg, 0.313ml, 1eq, 2 mmol) in DCM (30ml) at 0 °C. After 20 min, trichloroacetyl chloride (364 mg, 0.224 ml, 1 eq, 2 mmol) was added dropwise through a dropping funnel. The reaction mixture was allowed to warm to room temperature. After 4 h, the reaction was quenched with saturated NH_4Cl and partitioned between saturated $NaHCO_3$ and DCM. The organic layer was collected, and the aqueous layer was extracted using DCM (2 × 50 mL). The combined organic extracts were dried over $MgSO_4$, filtered, and concentrated *in vacuo* to yield product as a mixture of rotamers. The compound was purified by flash chromatography on silica gel with a mixture of hexane/EtOAc (85:15) as eluent. The solvent was removed *in vacuo* and the compound was characterised by NMR spectroscopy and compared with data reported before.[4]

Yield 467 mg (80%), yellow oil

^{1}H NMR (400 MHz, Chloroform-*d*) δ 7.63 – 7.15 (m, 5H), 5.26 (s, 2H), 4.85 (d, *J* = 119.9 Hz, 2H), 4.14 (d, *J* = 135.9 Hz, 2H).

^{13}C NMR (101 MHz, Chloroform-*d*) δ 160.86, 132.01, 128.84, 127.87, 127.17, 52.39, 51.27, 50.03.

1-benzyl-3,3-dichloro-4-(chloromethyl)pyrrolidin-2-one (7`)

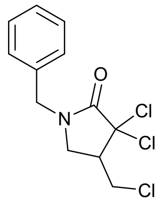

1-benzyl-3,3-dichloro-4-(chloromethyl)pyrrolidin-2-one

The reaction was performed under inert atmosphere with oven dried glassware. α-halo-amide substrate (0.2 mmol, 58.5 mg, 1 eq), CuCl (6 mg, 0.06 mmol, 0.3 eq), TPMA (17.4 mg, 0.06 mmol, 0.3 eq) and a magnetic stir bar was added to the reaction vial. The reaction was sealed, purged with argon, then 5 ml anhydrous toluene were added. The mixture was stirred under heating in an oil bath (80 °C). After reaction completion according to TLC, the solution was filtered through a silica plug, concentrated *in vacuo* to give the crude product which was purified on a flash silica column with mixture of hexane/EtOAc (60:40) as eluent. The solvent was removed *in vacuo* and the compound was characterised by NMR spectroscopy and compared with data reported before.[4] A calibration curve for GC was measured with benzodioxol as internal standard.

Yield 24 mg (41%), white powder

^{1}H NMR (400 MHz, Chloroform-*d*) δ 7.43 – 7.30 (m, 2H), 7.31 – 7.22 (m, 3H), 4.66 (d, *J* = 14.6 Hz, 1H), 4.48 (d, *J* = 14.7 Hz, 1H), 4.00 (dd, *J* = 11.3, 3.9 Hz, 1H), 3.71 (d, *J* = 9.9 Hz, 1H), 3.49 (d, *J* = 3.2 Hz, 1H), 3.21 – 3.03 (m, 2H).

^{13}C NMR (101 MHz, Chloroform-*d*) δ 166.04, 134.52, 129.07, 128.28, 51.62, 47.90, 47.27, 41.03.

GC calibration curve of substate 7`:

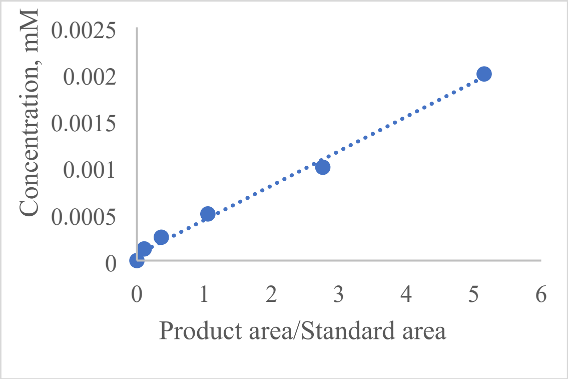

N-benzyl-2-bromo-N-(but-3-en-1-yl)-2-methylpropanamide(8)

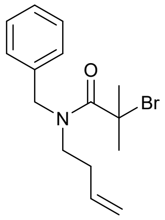

N-benzyl-2-bromo-N-(but-3-en-1-yl)-2-methylpropanamide

Triethylamine (0.42ml, 1.5 eq, 3 mmol) was added to a solution of N-benzylbut-3-en-1-amine (323 mg, 1 eq, 2 mmol) in DCM (15ml) at 0 °C. After 20 min, α-bromoisobuturyl bromide (460mg, 0.25 ml,

1 eq, 2 mmol) was added dropwise through a dropping funnel. The reaction mixture was allowed to warm to room temperature. After 4 h, the reaction was quenched with saturated NH_4Cl and partitioned between saturated $NaHCO_3$ and DCM. The organic layer was collected, and the aqueous layer was extracted using DCM (2 × 50 mL). The combined organic extracts were dried over $MgSO_4$, filtered, and concentrated *in vacuo* to yield the product. The compound was purified by flash chromatography on silica gel with a mixture of hexane/EtOAc (75:25) as eluent. The solvent was removed *in vacuo* and the compound was characterised by NMR spectroscopy

Yield 400 mg (64%), colorless oil

^{1}H NMR (400 MHz, Chloroform-*d*) δ 7.45 – 7.13 (m, 5H), 5.74 (ddt, *J* = 17.1, 10.2, 6.9 Hz, 1H), 5.25 – 4.45 (m, 4H), 3.52 (d, *J* = 137.7 Hz, 2H), 2.35 (s, 2H), 1.99 (s, 6H)

1-benzyl-4-(bromomethyl)-3,3-dimethylpiperidin-2-one(8`)

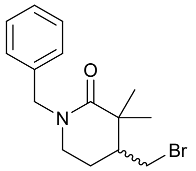

1-benzyl-4-(bromomethyl)-3,3-dimethylpiperidin-2-one

The reaction was performed under inert atmosphere with oven dried glassware. α-halo-amide substrate (0.1 mmol, 31 mg, 1 eq), CuBr (14.3 mg, 0.1 mmol, 1 eq), TPMA (29 mg, 0.1 mmol, 1 eq) and a magnetic stir bar was added to the reaction vial. The reaction was sealed, purged with argon, then 5 ml anhydrous toluene were added. The mixture was stirred under heating in an oil bath (80 °C). After reaction completion according to TLC, the solution was filtered through a silica plug, concentrated *in vacuo* to give the crude product which was purified on a flash silica column with mixture of hexane/EtOAc (60:40) as eluent. The solvent was removed *in vacuo* and the compound was characterised by NMR spectroscopy and compared with data reported before.[4] A calibration curve for GC was measured with acetophenone as internal standard.

Yield

^{1}H NMR (400 MHz, Chloroform-*d*) δ 7.42 – 7.10 (m, 6H), 4.62 (d, *J* = 14.5 Hz, 1H), 4.50 (d, *J* = 14.6 Hz, 1H), 3.65 (dd, *J* = 10.1, 3.1 Hz, 1H), 3.32 – 3.21 (m, 2H), 3.14 (dd, *J* = 11.1, 10.1 Hz, 1H), 2.22 (dtd, *J* = 13.7, 4.5, 2.9 Hz, 1H), 2.09 – 2.00 (m, 1H), 1.83 – 1.66 (m, 1H), 1.38 (s, 3H), 1.16 (s, 3H).

^{13}C NMR (101 MHz, Chloroform-*d*) δ 174.64, 137.30, 128.65, 127.92, 127.39, 50.71, 45.89, 45.58, 42.45, 34.21, 26.22, 22.89, 21.33.

GC calibration curve of substate **8**`:

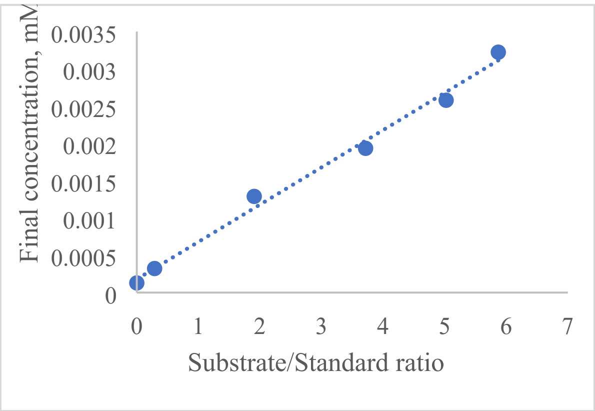

N,N,N',N'-tetrabenzylmethanediamine

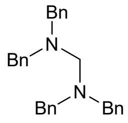

N,N,N',N'-tetrabenzylmethanediamine

The following procedure was adapted from Kaiser et al.[5]To a round-bottom flask charged with 10 g of dibenzylamine (52mmol) and a magnetic stir-bar at 0 °C, 2 ml of an aqueous solution of formaldehyde (37%) was added dropwise and the resulting biphasic mixture was stirred vigorously at ambient temperature (23°C) for 12 h. The reaction resulted in white solid, that was dissolved in ethyl acetate and washed with brine. The organic phases were combined, dried over anhydrous $NaSO_4$, concentrated under reduced pressure, and used in further steps without any other treatment.

(Z)-N-benzyl-3-cyclopropylprop-2-en-1-amine

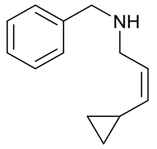

(Z)-N-benzyl-3-cyclopropylprop-2-en-1-amine

The following procedure was adapted from Kaiser et al.[5] A round-bottom flask charged with N,N,N',N'-tetrabenzyldiaminomethane (2.3g, 5.8mmol) and a magnetic stir-bar was cooled to 0 °C under argon-atmosphere and 5ml of dichloroethane was added. Afterward, trifluoroacetic acid (0.36ml, 4.8mmol) and cyclopropylacetylene(317mg, 4.80.5 mmol) were added in one portion, the flask was heated in an oil bath at 75 °C. The reaction was vigorously stirred at this temperature for 15 h, after which it was allowed to cool to room temperature. Subsequently, volatile components were removed under reduced pressure. The combined organic phases were then dried over anhydrous $NaSO_4$ and filtered. The filtrate was concentrated under reduced pressure to afford the crude product, which was purified by flash column chromatography on basified silica gel (Hexane/EtOAc: 80:20) to afford the analytically pure product.

Yield 320 mg (30%), colorless oil

^{1}H NMR ($CDCl_3$, 400 MHz): δ 7.31 (d, J = 4.4 Hz, 5H), 7.25 – 7.19 (m, 1H), 5.63 (dt, J = 15.3, 6.4 Hz, 1H), 5.15 (ddt, J = 15.3, 8.7, 1.4 Hz, 1H), 3.77 (s, 2H), 3.20 (dd, J = 6.4, 1.4 Hz, 2H), 0.73 – 0.60 (m, 2H), 0.34 (dt, J = 6.4, 4.4 Hz, 2H).

^{13}C NMR (101 MHz, Chloroform-*d*) δ 140.37, 136.46, 128.37, 128.19, 126.89, 125.82, 53.28, 51.05, 13.43, 6.59.

Supplementary Data

Supplementary Tables

Table SI1. Total turnover number (TTN) for the transformation of **1** to **2** with different reaction conditions.

Entry	Reaction conditions and deviations	TTN
1	Purified protein	62±6
2	Purified protein without sodium ascorbate	<1
3	Purified protein + open flask	<1
4	Purified protein without sodium ascorbate with glucose	<1
5	No myoglobin	**2** not observed
6	Cofactor only (0.1 mol%)	36±3

Reaction conditions (unless otherwise stated): 6 µmol **1**, 6 nmol H93S variant, 5 eq. sodium ascorbate, 40°C, 16 h, phosphate buffer (50 mM, 250 mM NaCl, pH 7.4), 3% DMSO, inert atmosphere

Table SI2 Total turnover number (TTN) for the transformation of **1** to **2** using whole cells or cell lysate with different reaction conditions.

Entry	Reaction conditions and deviations	TTN
1	Whole cell	9±1
2	Whole cell + open flask	<1
3	Whole cell + 5 eq. sodium ascorbate	29±8
4	Whole cell + 5 eq. glucose	101±13
5	Lysate	94±11
6	Lysate + 5 eq. sodium ascorbate	68±7
7	Lysate + 5 eq. glucose	109±7

Reaction conditions (unless otherwise stated): 6 µmol **1**, 0.5 ml whole cells containing H93S at OD(600) = 40, 22°C, 16 h, sodium phosphate buffer (100 mM, pH 7.4), 10% DMSO, inert atmosphere

Table SI3 Yield for the transformation of **1** to **2** in Whole cells catalysis.

Entry	Reaction conditions and deviations	Yield, %
1	Whole cells(Dh5alpha) without myoglobin	2
2	Whole cells (Dh5alpha) without myoglobin, with addition of ALA	7
3	Whole cells(BL21) expressing H93S myoglobin	20

Reaction conditions (unless otherwise stated): 30 mM **1**, 0.5 ml whole cells containing H93S at OD(600) = 40, 22°C, 16 h, sodium phosphate buffer (100 mM, pH 7.4), 10% DMSO, inert atmosphere

Supplementary Figures

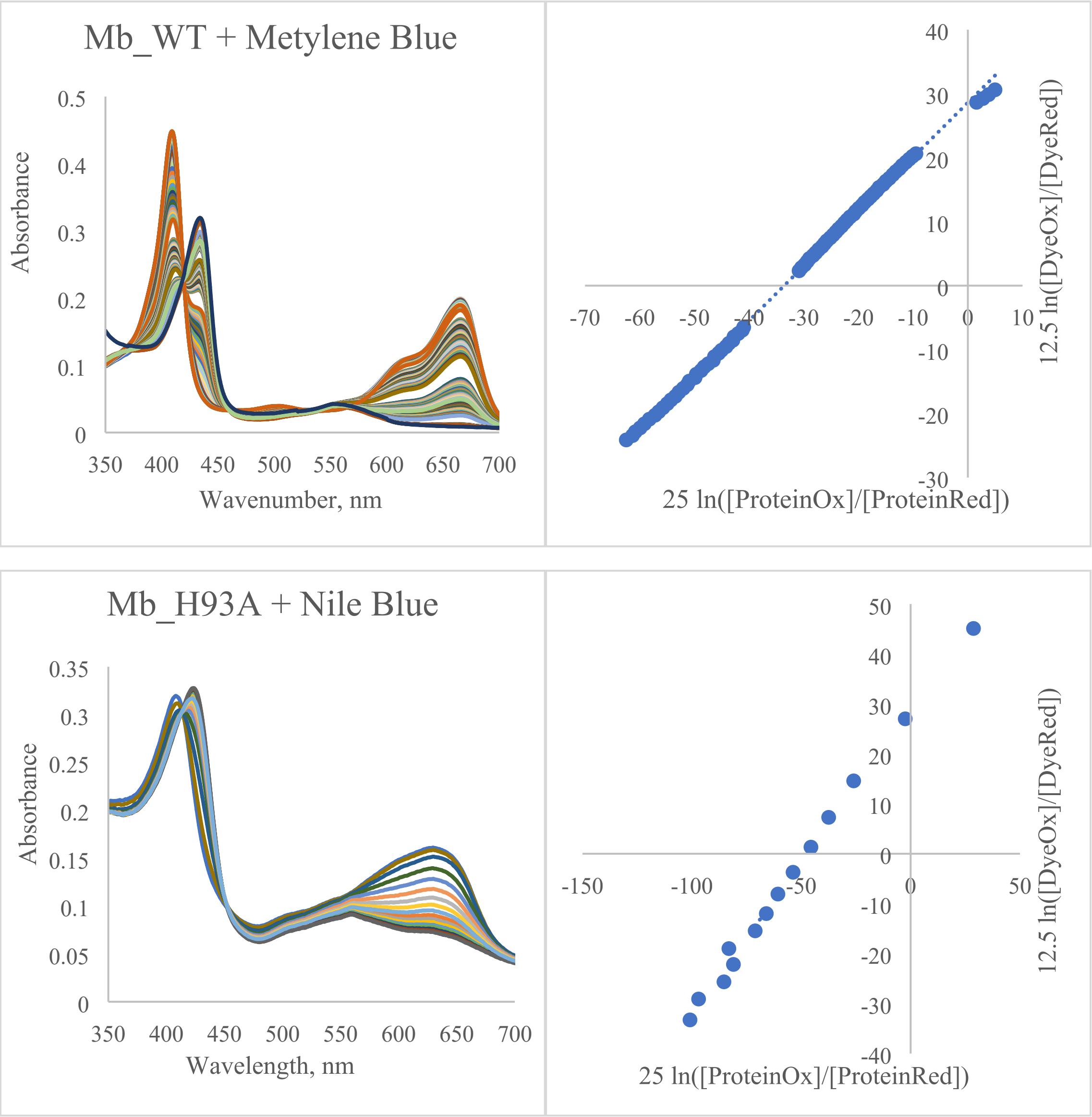

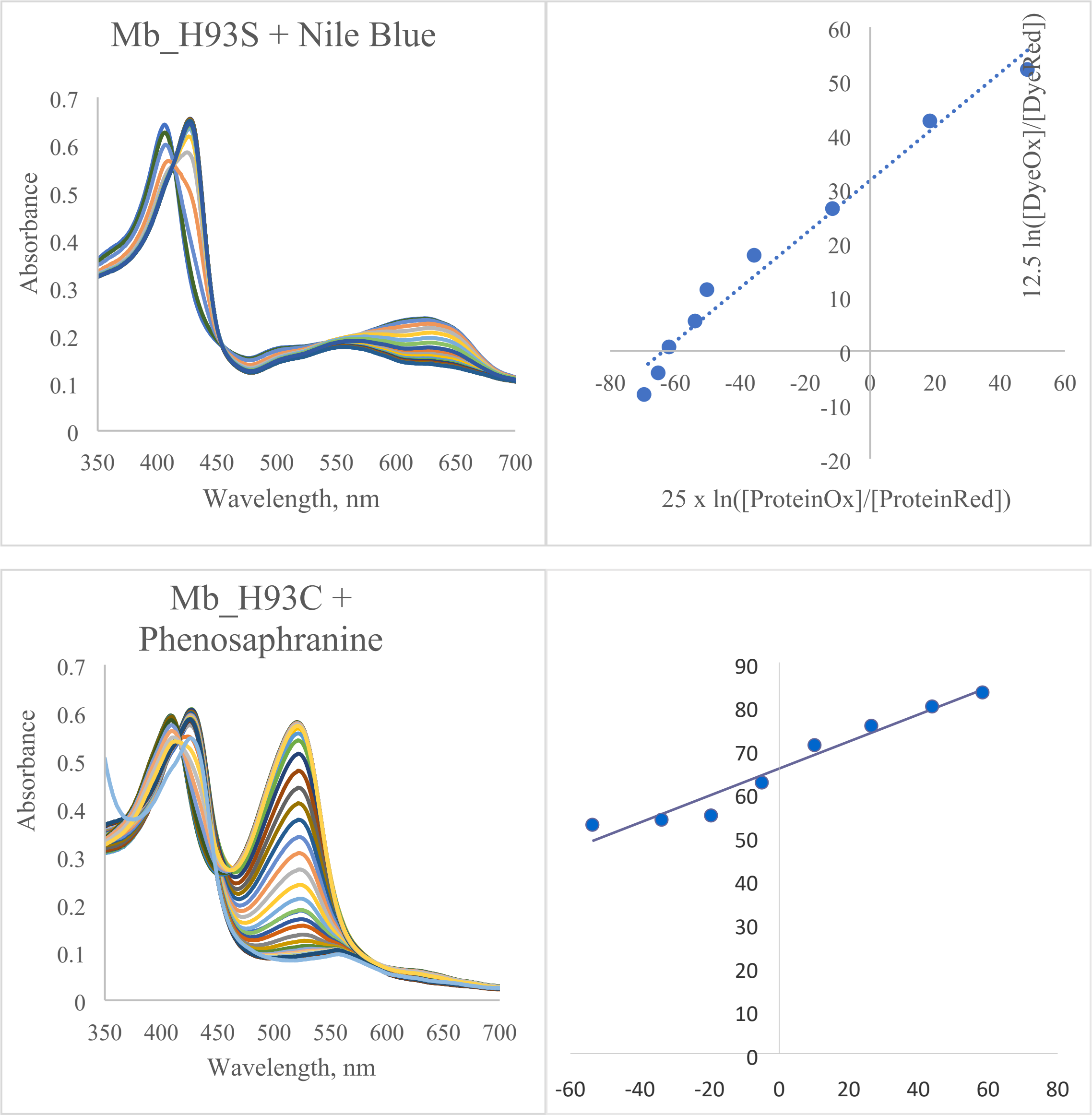

Myoglobin variant	Dye/Redox potential, mV	Intercept	Redox potential of a myoglobin variant, mV
WT	Methylene blue/11	29	40
H93A	Nile Blue/ -116	29	-87
H93S	Nile Blue/ -116	32	-84
H93C	Phenosaphranine/ - 252	66	-186

Figure SI1. Redox potential measurements of myoglobin variants. The redox potential was calculated as sum of redox potential of the dye and graph intercept of y-axis

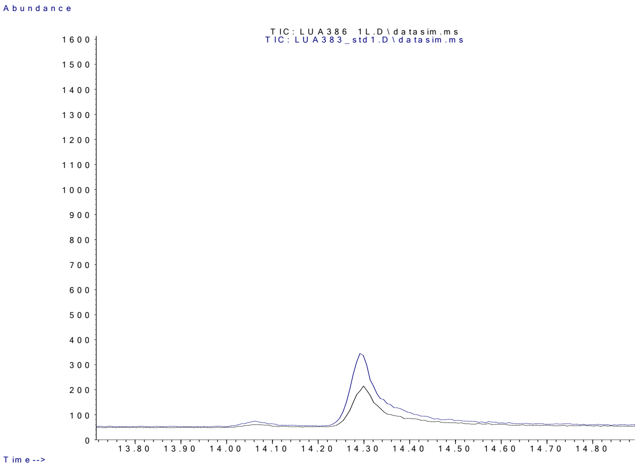

Figure SI2. Overlay of gas chromatograms of cyclised model compound 1-benzyl-4-(4-bromobut-1-en-1-yl)-3,3-dimethylpyrrolidin-2-one (blue) and cyclisation result(black) after reaction with myoglobin H93S variant (30 mM **3**, 0.1 mol% Myoglobin H93S, 5 eq. sodium ascorbate, 40°C, 16 h, phosphate buffer (50 mM, 250 mM NaCl, pH 7.4), 3 vol% DMSO, inert atmosphere).

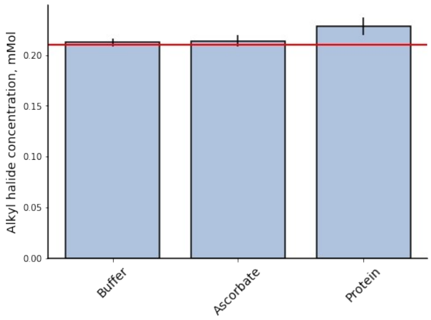

Figure SI3. Stability of alkyl halide 2 in various reaction conditions. : 0.21 µmol l **2**, 4.5 nmol H93S variant(if present), 30 mmol sodium ascorbate(if present), 40°C, 0.3ml phosphate buffer (50 mM, 250 mM NaCl, pH 7.4), 3% DMSO, inert atmosphere. n = 2

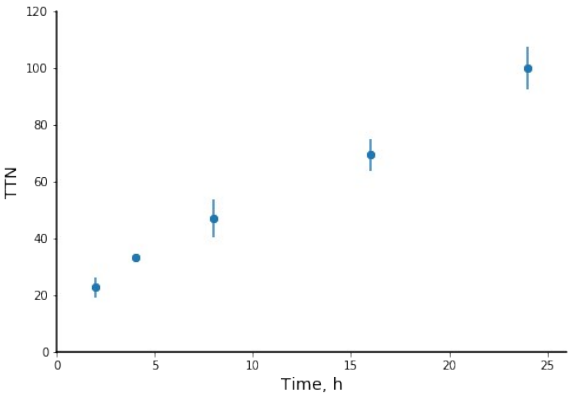

Figure SI4. Plot of TTN of conversion of 1 to 2 vs time. Reaction conditions : 6 µmol l **1**, 4.5 nmol H93S variant, 30 mmol sodium ascorbate, 40°C, 0.3ml phosphate buffer (50 mM, 250 mM NaCl, pH 7.4), 3% DMSO, inert atmosphere. n = 2

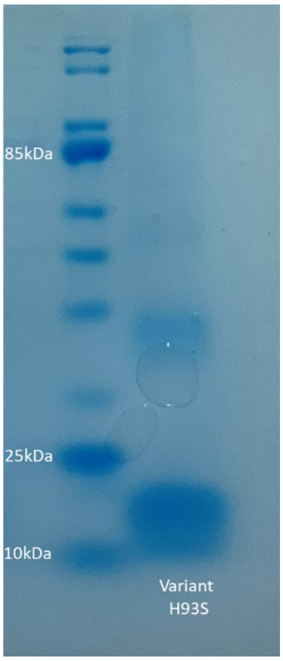

Figure SI5. Gel electrophoresis of myoglobin variant H93S

Chromatogram

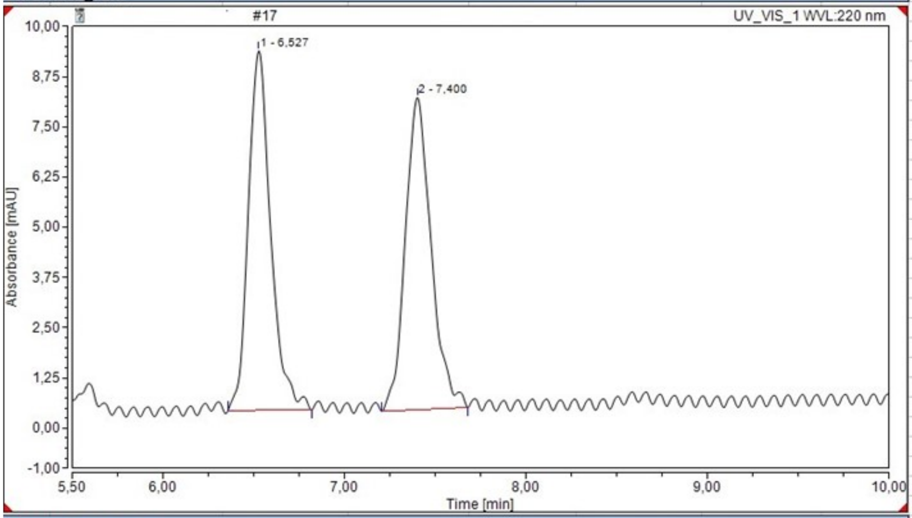

Integration Results

No.	Peak Name	Retention Time min	Area mAU*min	Height mAU	Relative Area %	Relative Height %	Amount n.a.
1		6,527	1,283	8,954	49,87	53,53	n.a.
2		7,400	1,289	7,774	50,13	46,47	n.a.
**Total:**			**2,572**	**16,728**	**100,00**	**100,00**	

Chromatogram

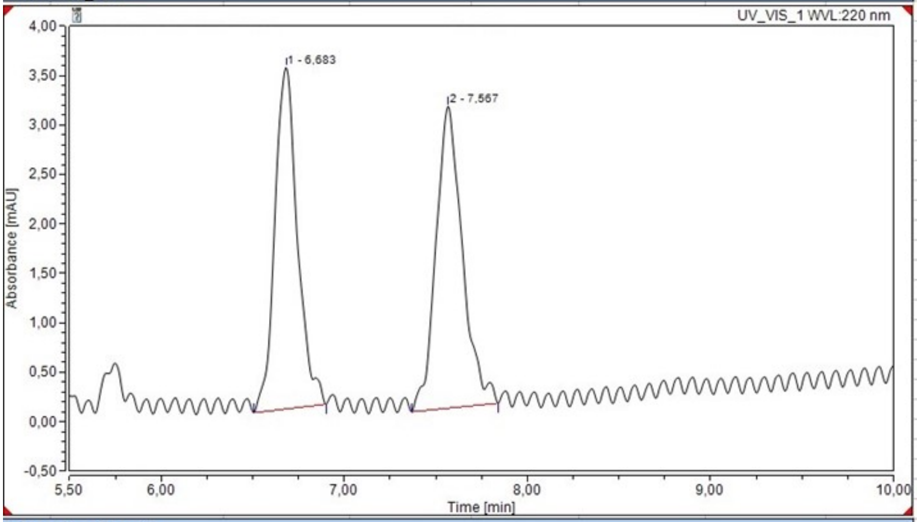

Integration Results

No.	Peak Name	Retention Time min	Area mAU*min	Height mAU	Relative Area %	Relative Height %	Amount n.a.
1		6,683	0,495	3,452	49,09	53,06	n.a.
2		7,567	0,513	3,054	50,91	46,94	n.a.
**Total:**			**1,008**	**6,507**	**100,00**	**100,00**	

Fig SI6. LC chromatography of standard product **2** (up) and sample from the biocatalytic transformation of **1** to **2** (down). Run conditions: Chiralpak IH column (5µm particle size, 250 x 4.6 mm i.d.), 10 min Hexane/Ethanol (80:20) at 1ml/min

NMR Spectra

1H NMR spectra of N-allyl-N-benzyl-2-bromo-2-methylpropanamide (**1**) :

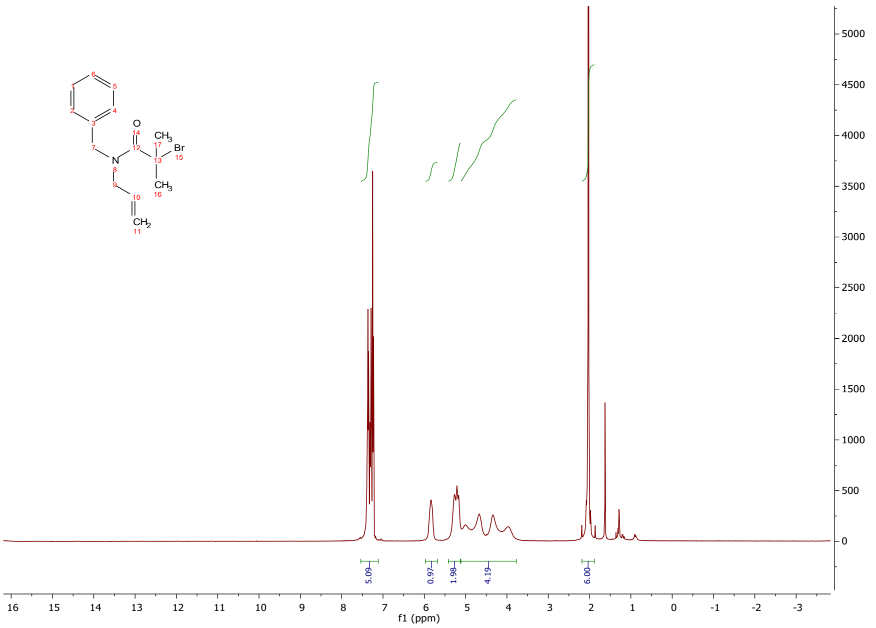

13C NMR spectra of N-allyl-N-benzyl-2-bromo-2-methylpropanamide (**1**) :

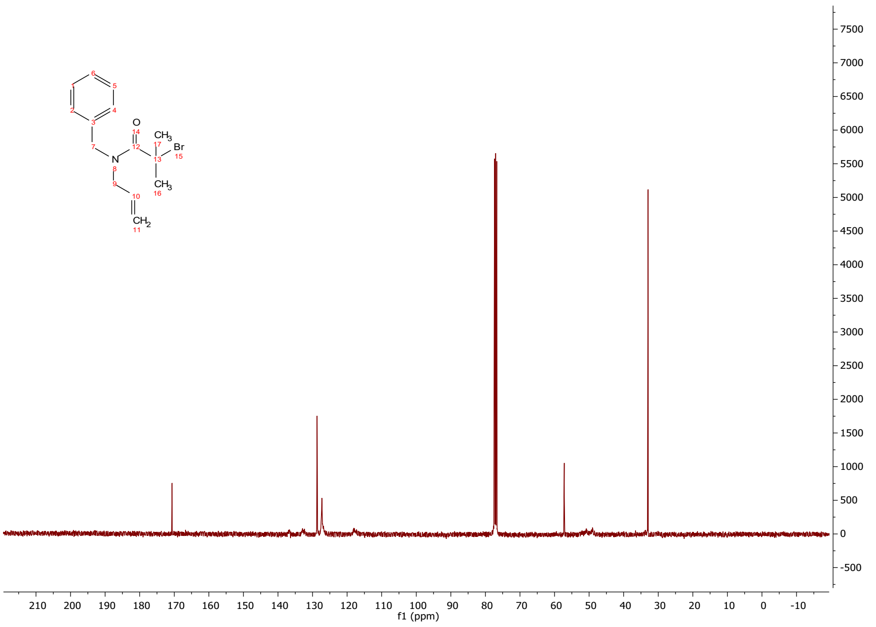

1H NMR spectra of N,N-diallyl-2,2,2-trichloroacetamide (**2**) :

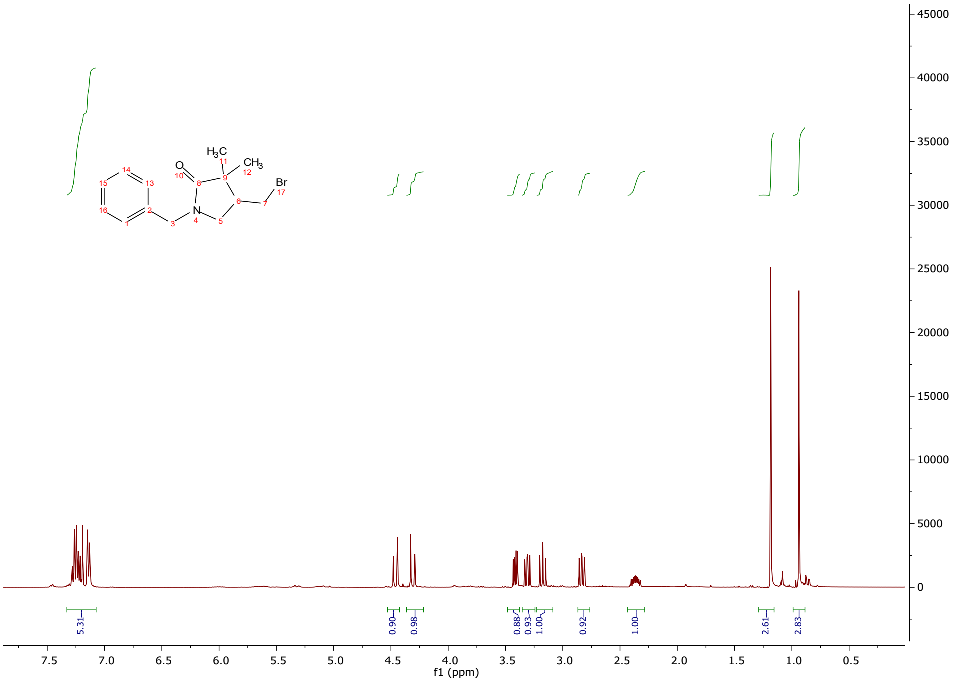

13C NMR spectra of N,N-diallyl-2,2,2-trichloroacetamide (**2**) :

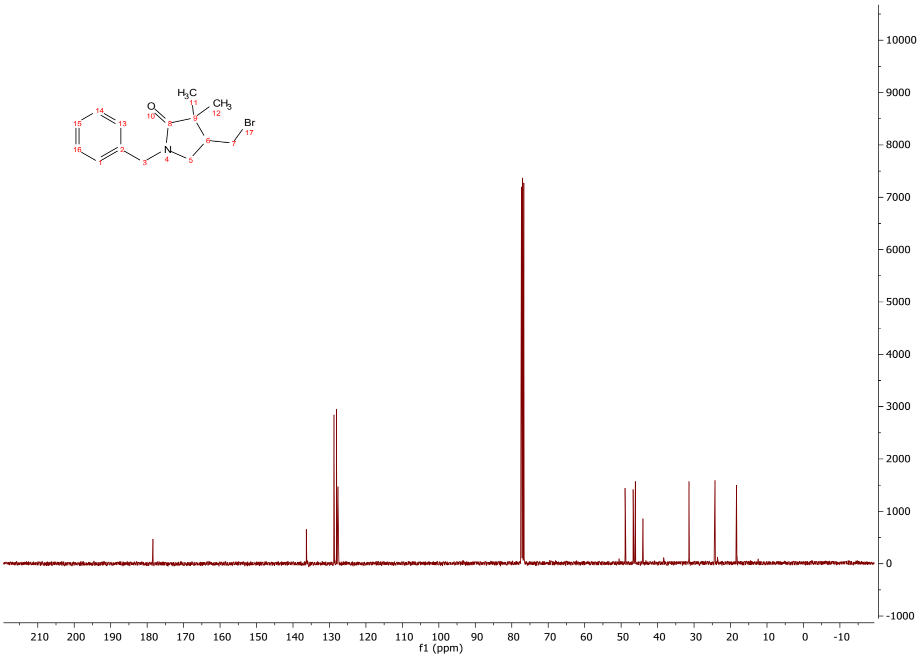

1H NMR spectra of N-benzyl-2-bromo-N-(3-cyclopropylallyl)-2-methylpropanamide (**3**) :

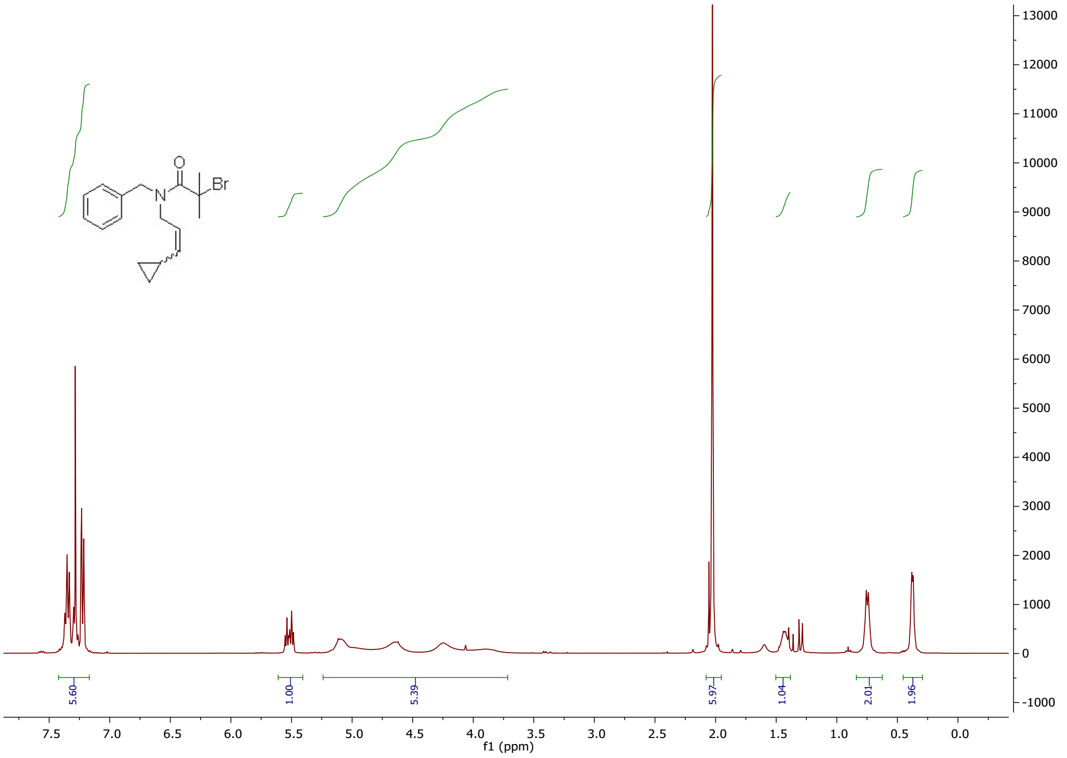

13C NMR spectra of N-benzyl-2-bromo-N-(3-cyclopropylallyl)-2-methylpropanamide (**3**) :

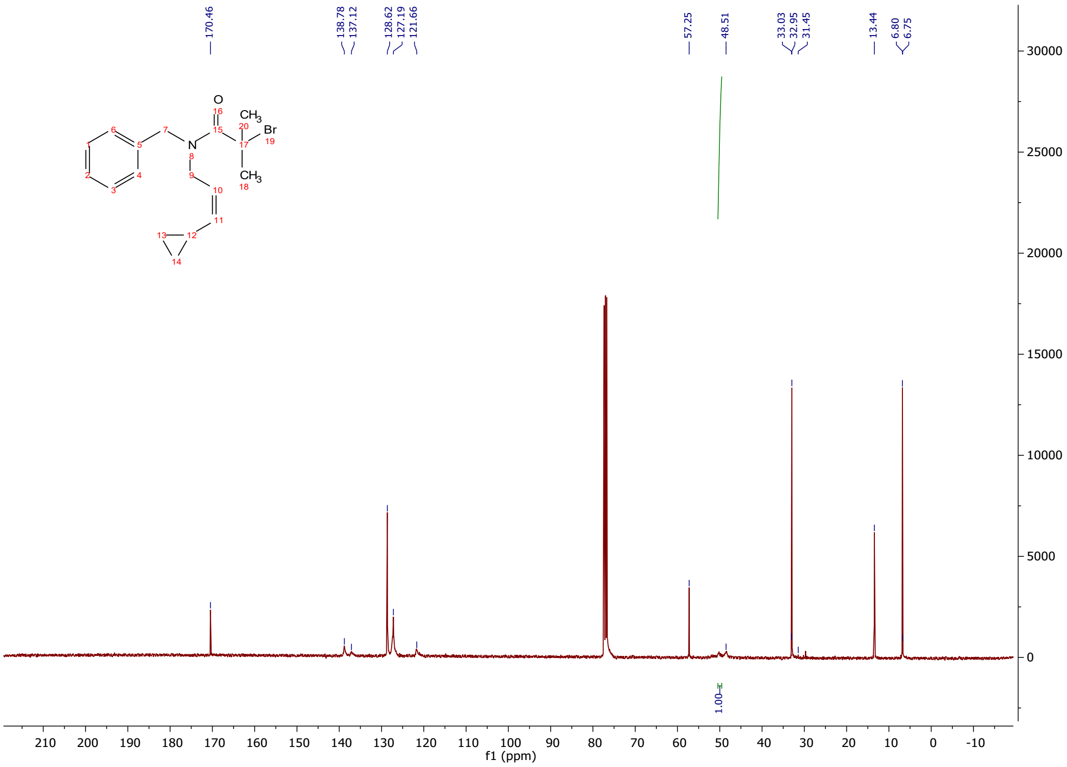

1H NMR spectra of N-benzyl-2-bromo-N-(3-cyclopropylallyl)-2-methylpropanamide (**4**) :

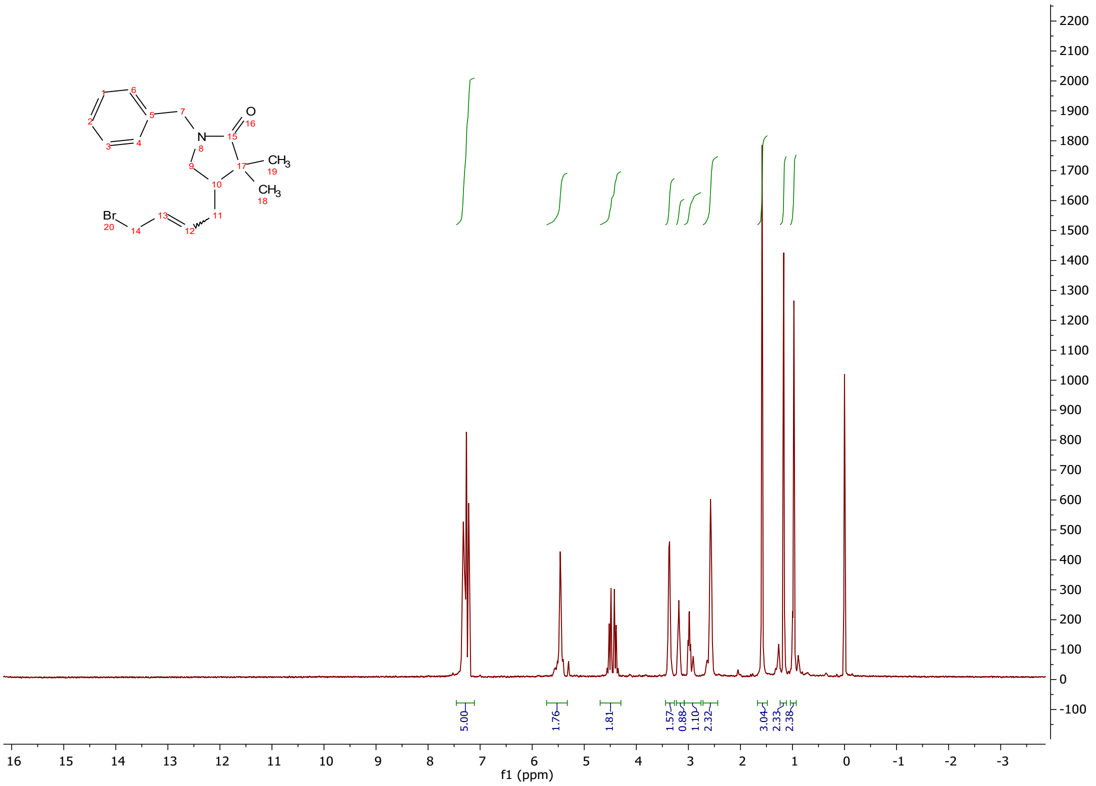

13C NMR spectra of N-benzyl-2-bromo-N-(3-cyclopropylallyl)-2-methylpropanamide (**4**) :

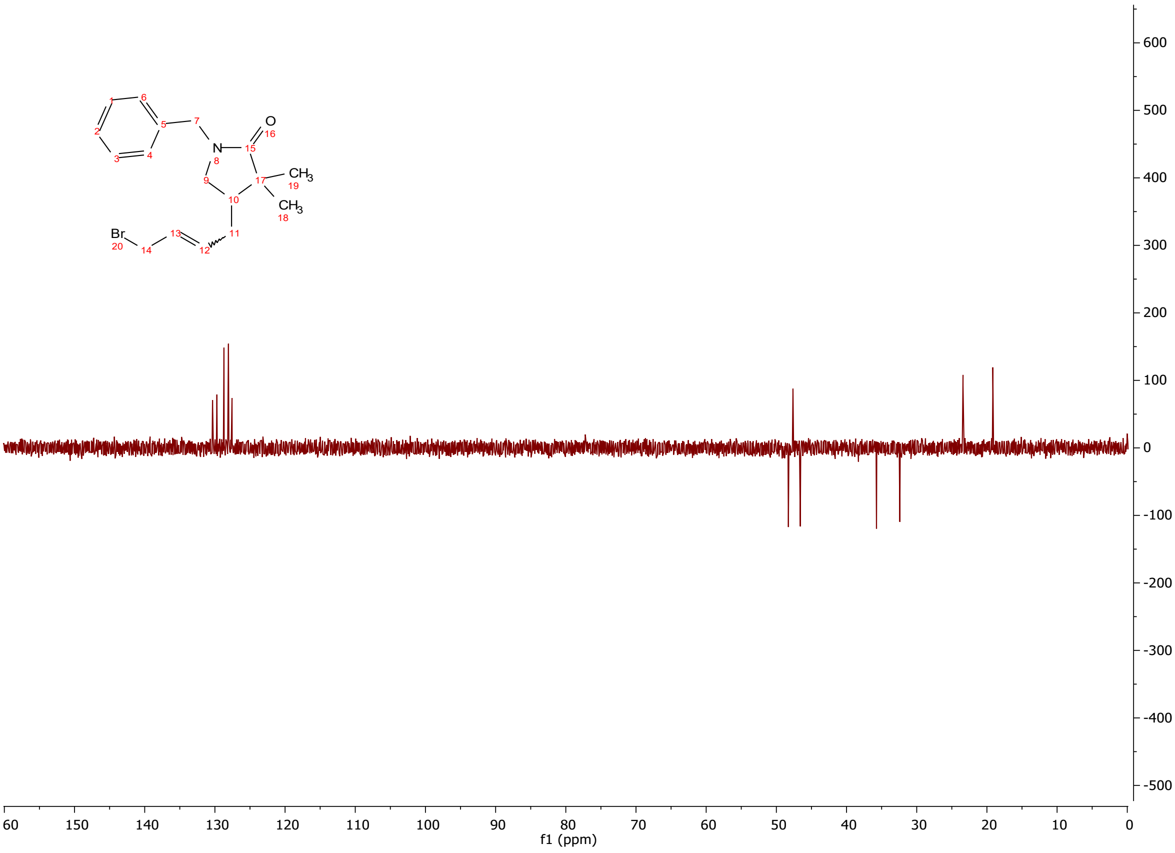

1H NMR spectra of N,N-diallyl-2-bromo-2-methylpropanamide (**5**) :

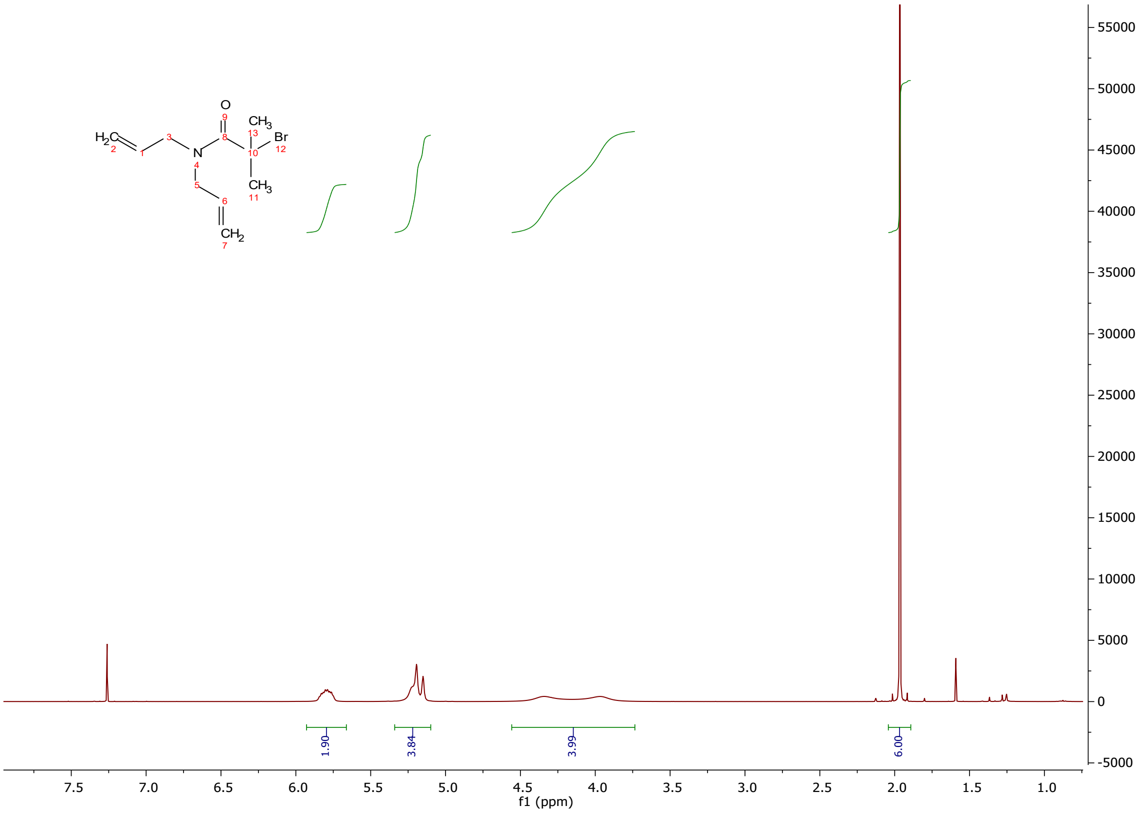

13C NMR spectra N,N-diallyl-2-bromo-2-methylpropanamide (**5**) :

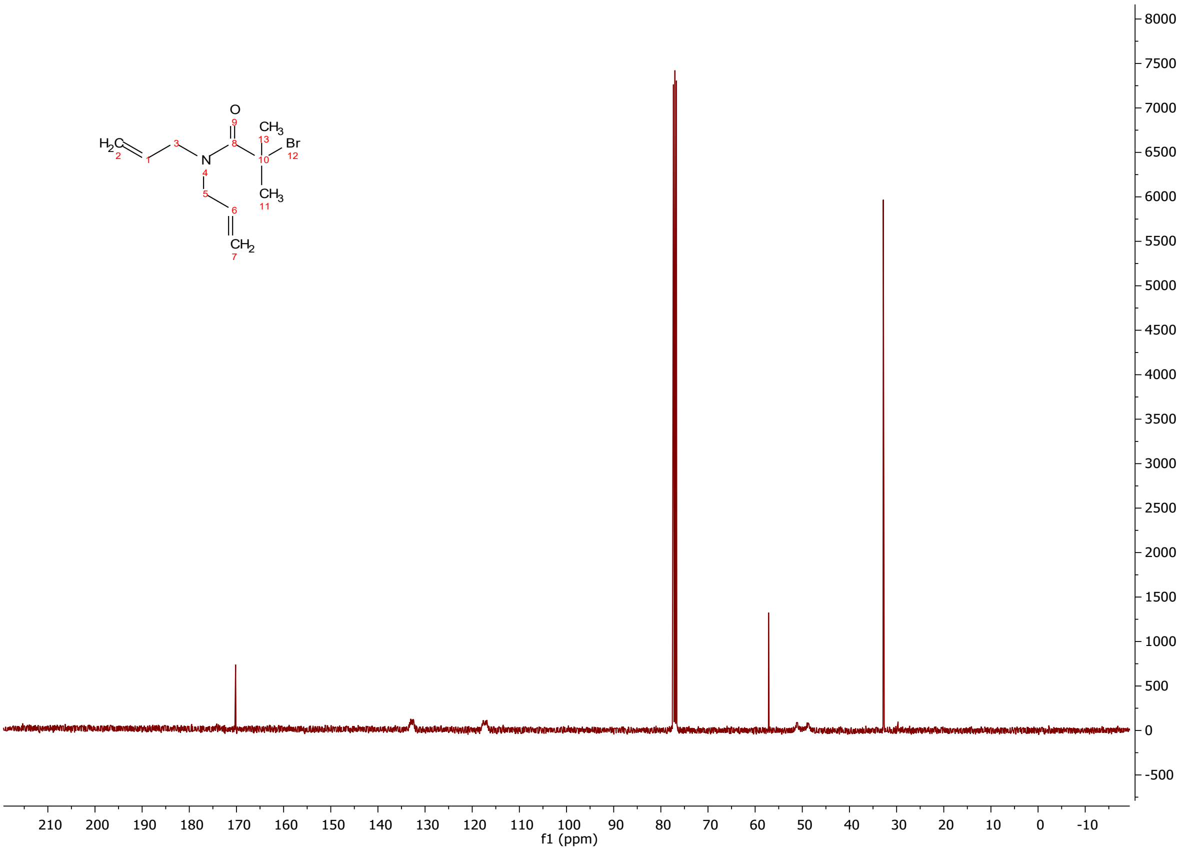

1H NMR spectra of 1-allyl-4-(bromomethyl)-3,3-dimethylpyrrolidin-2-one (**5`**) :

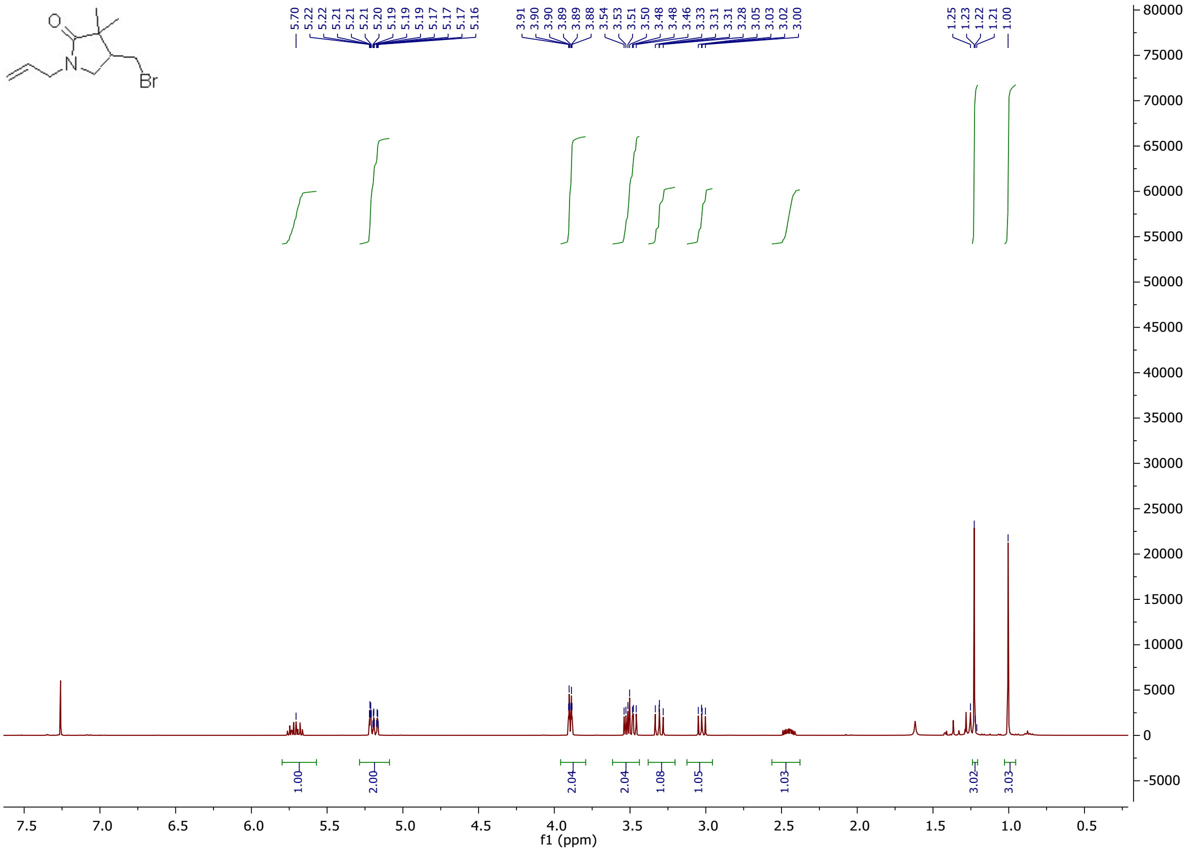

13C NMR spectra 1-allyl-4-(bromomethyl)-3,3-dimethylpyrrolidin-2-one (**5`**) :

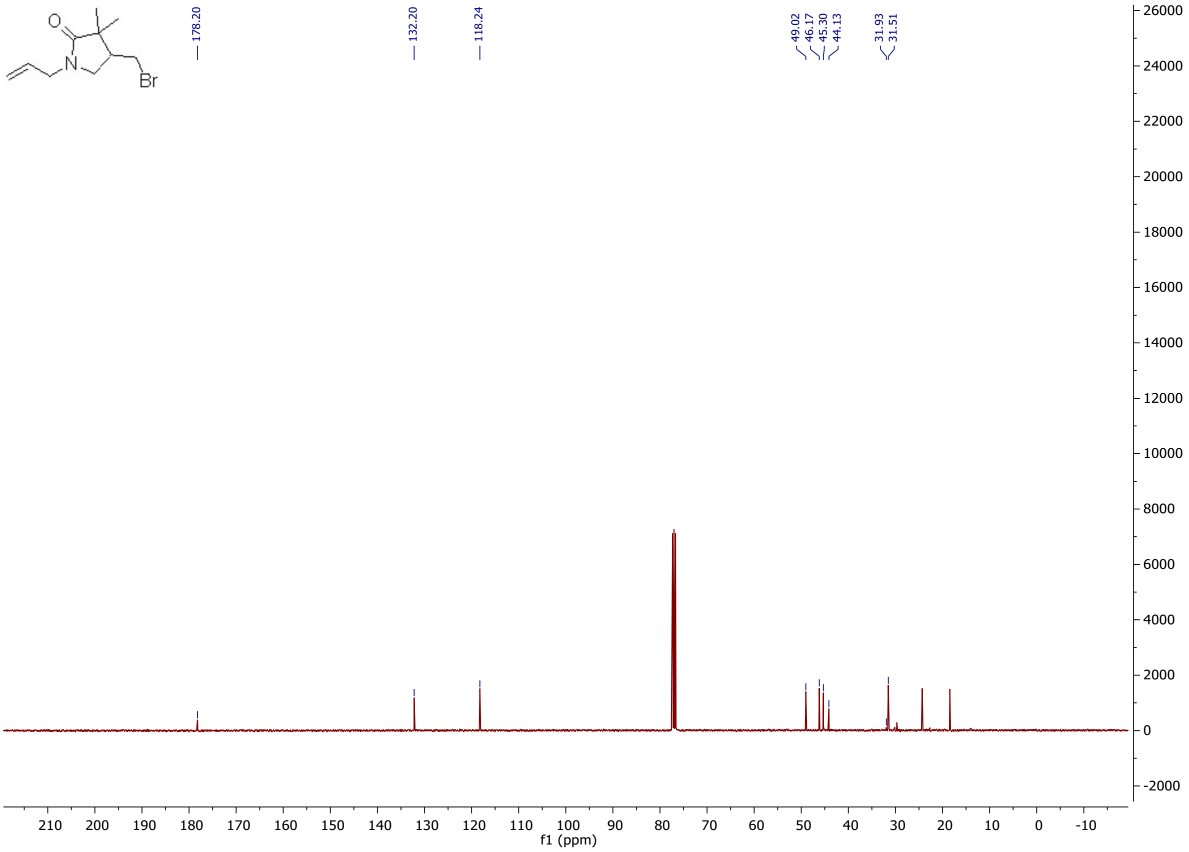

1H NMR spectra of N,N-diallyl-2,2,2-trichloroacetamide (**6**) :

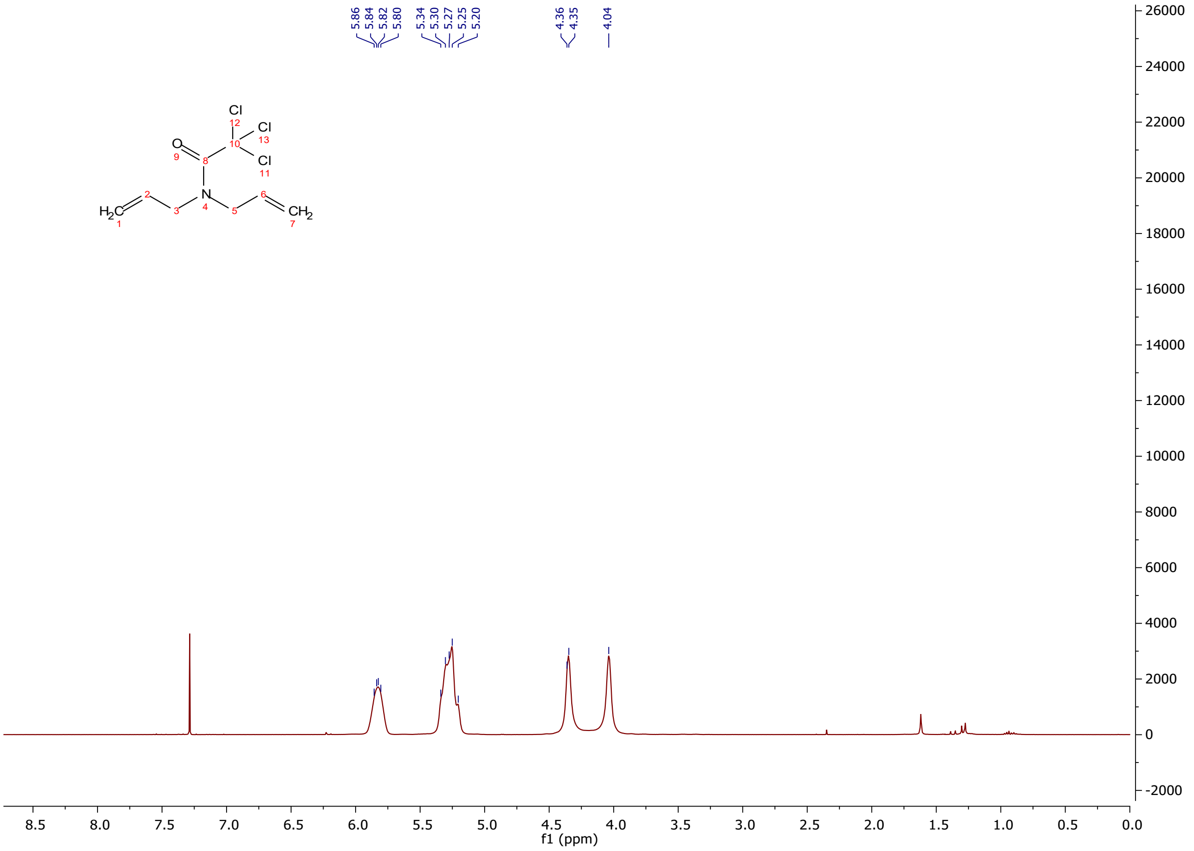

13C NMR spectra N,N-diallyl-2,2,2-trichloroacetamide (**6**) :

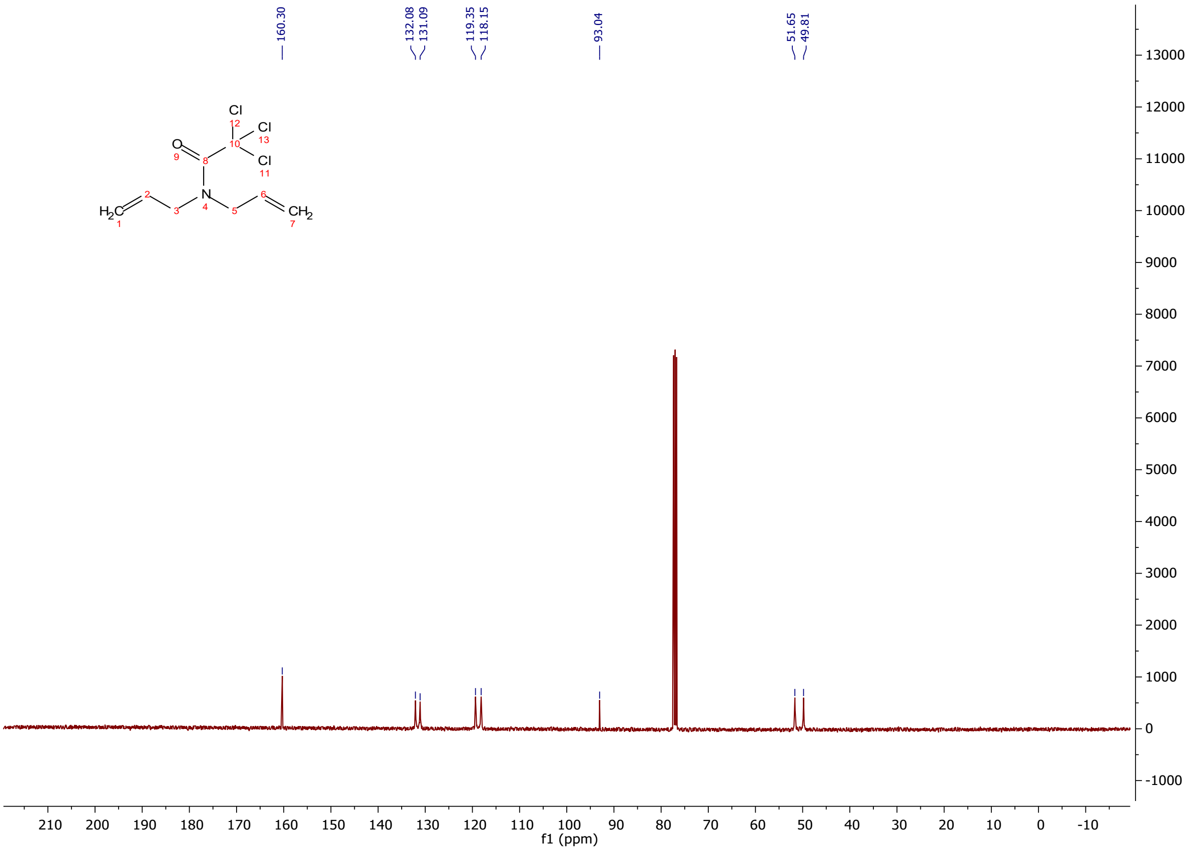

1H NMR spectra of 1-allyl-3,3-dichloro-4-(chloromethyl)pyrrolidin-2-one (**6`**) :

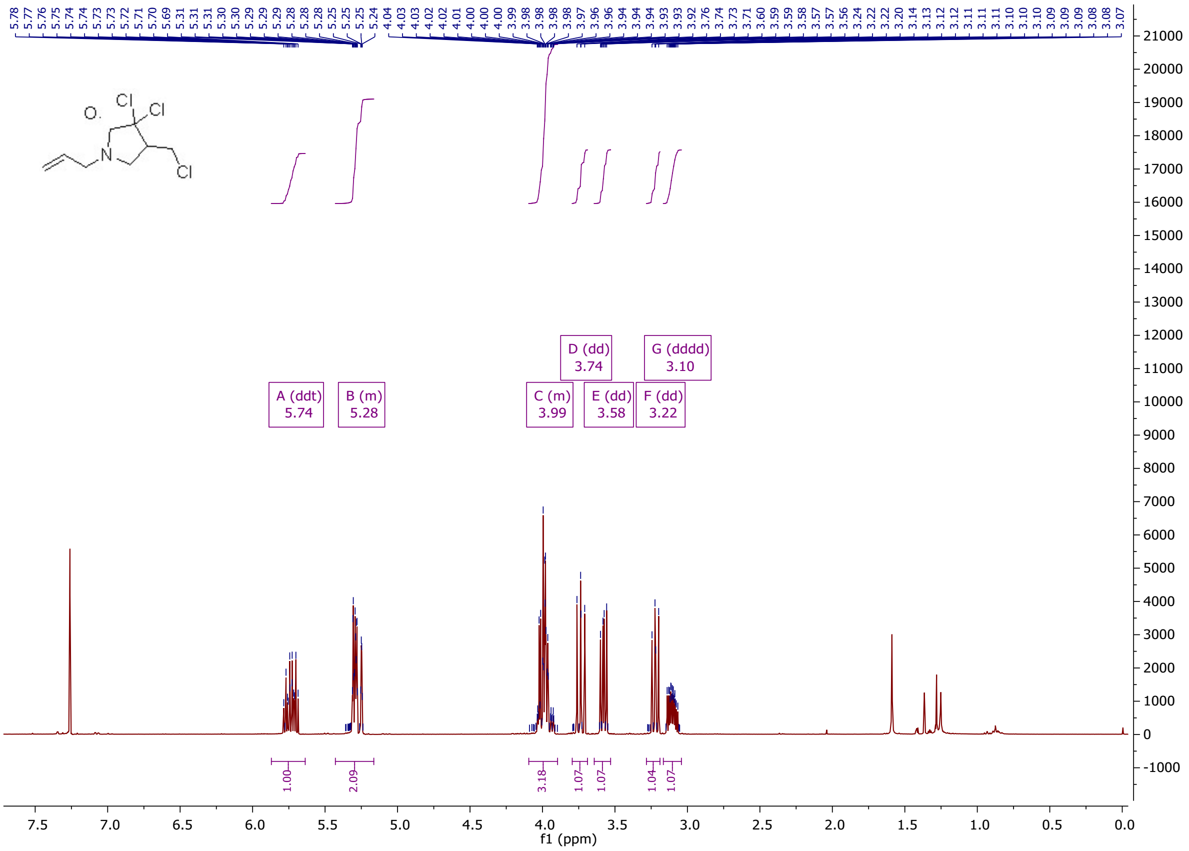

13C NMR spectra 1-allyl-3,3-dichloro-4-(chloromethyl)pyrrolidin-2-one (**6`**) :

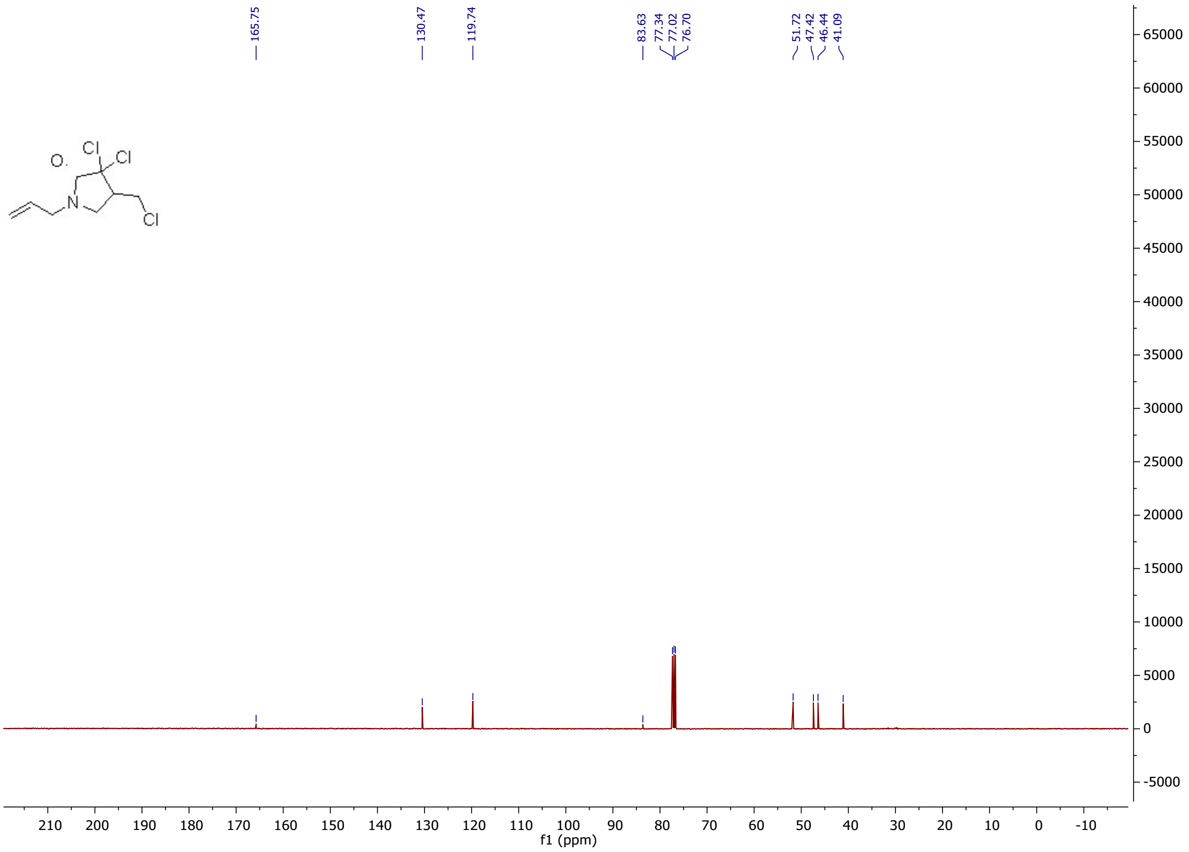

1H NMR spectra N-allyl-N-benzyl-2,2,2-trichloroacetamide (**7**) :

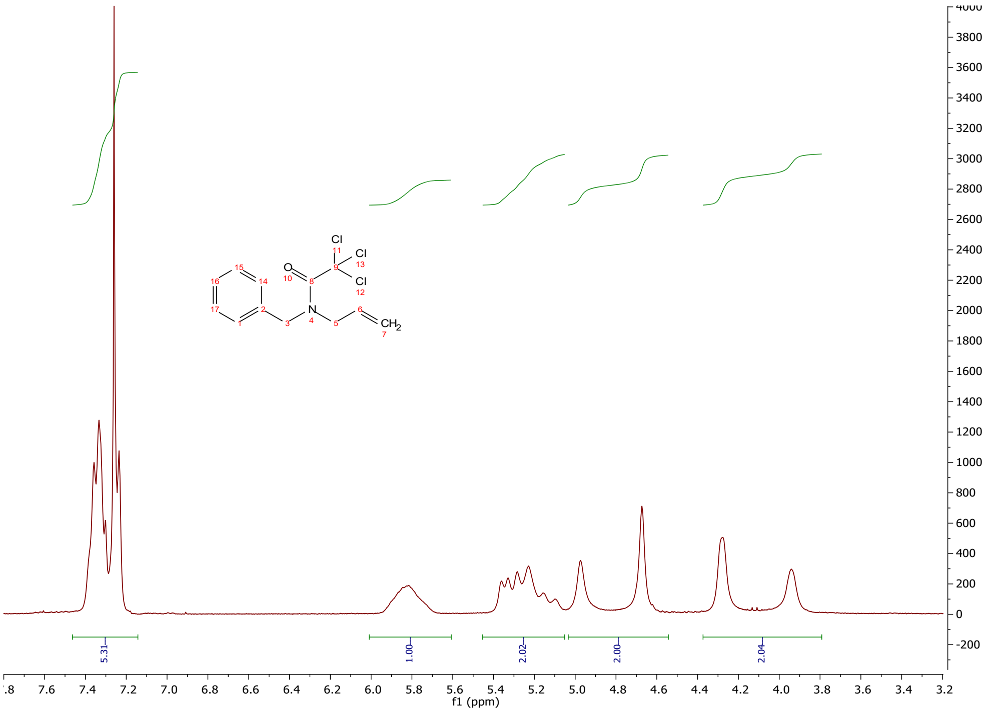

13C NMR spectra N-allyl-N-benzyl-2,2,2-trichloroacetamide (**7**) :

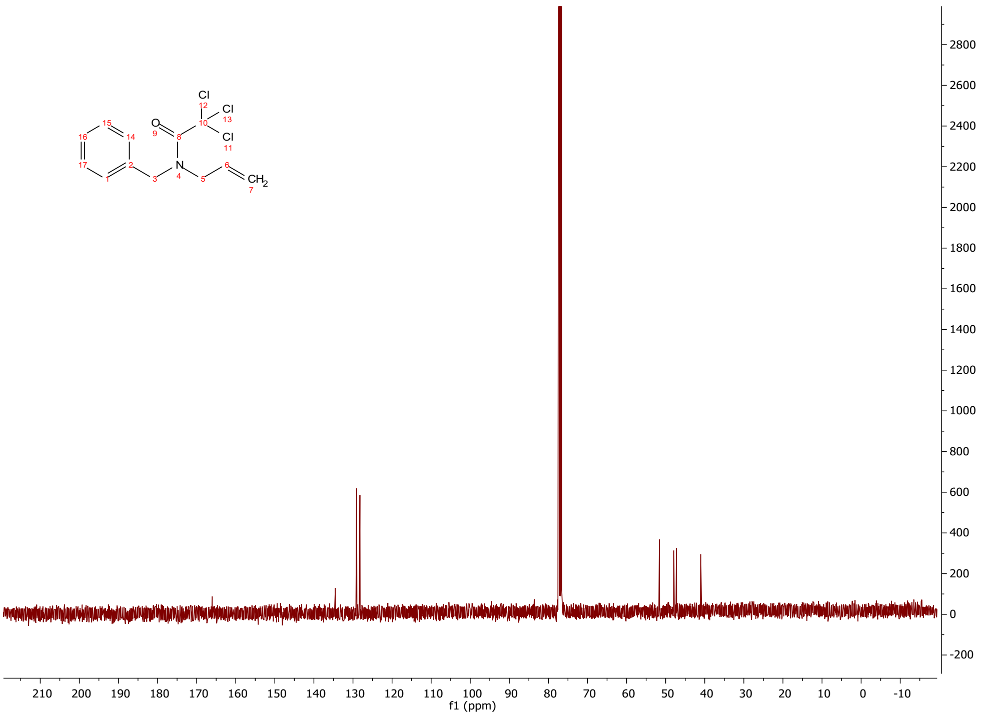

1H NMR spectra of 1-benzyl-3,3-dichloro-4-(chloromethyl)pyrrolidin-2-one (**7`**) :

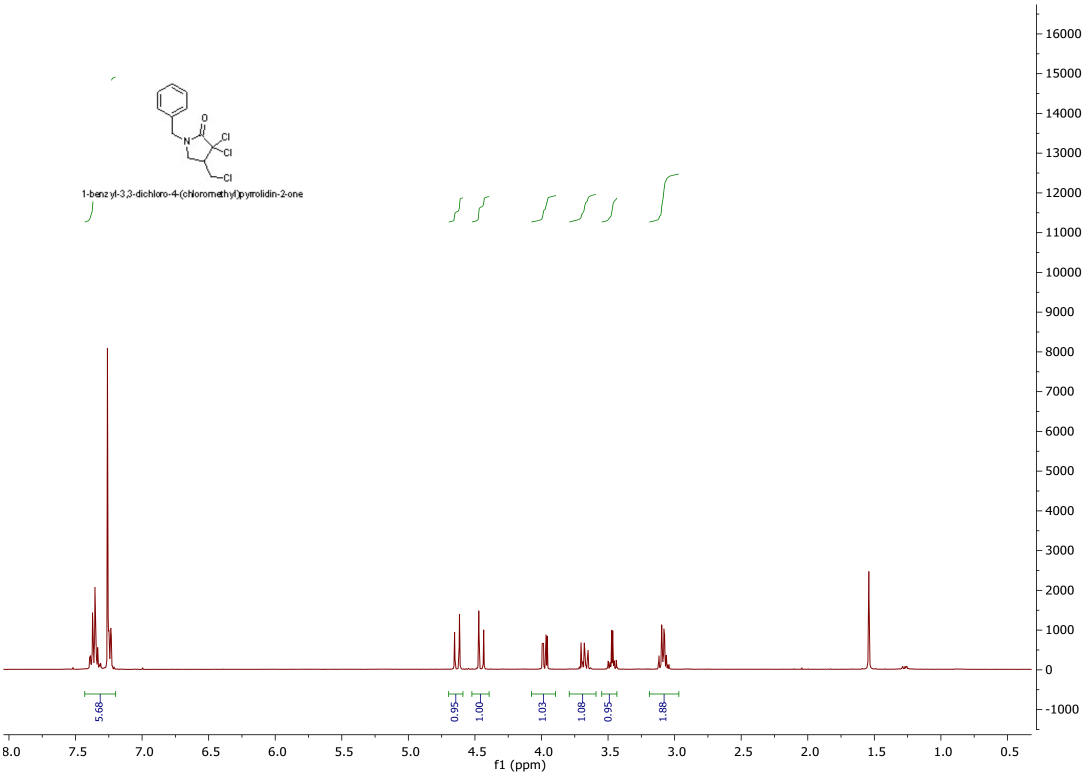

13C NMR spectra 1-benzyl-3,3-dichloro-4-(chloromethyl)pyrrolidin-2-one (**7`**) :

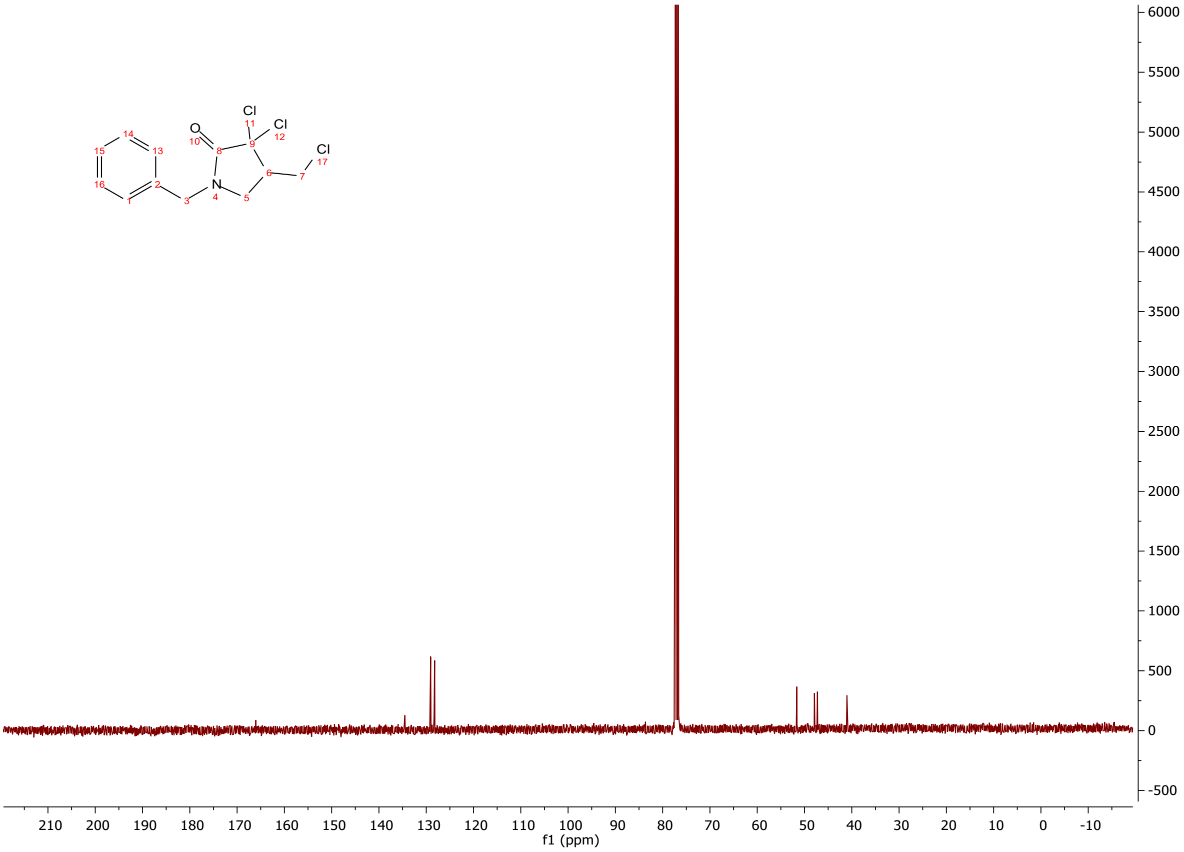

1H NMR spectra of N-benzyl-2-bromo-N-(but-3-en-1-yl)-2-methylpropanamide (**8**) :

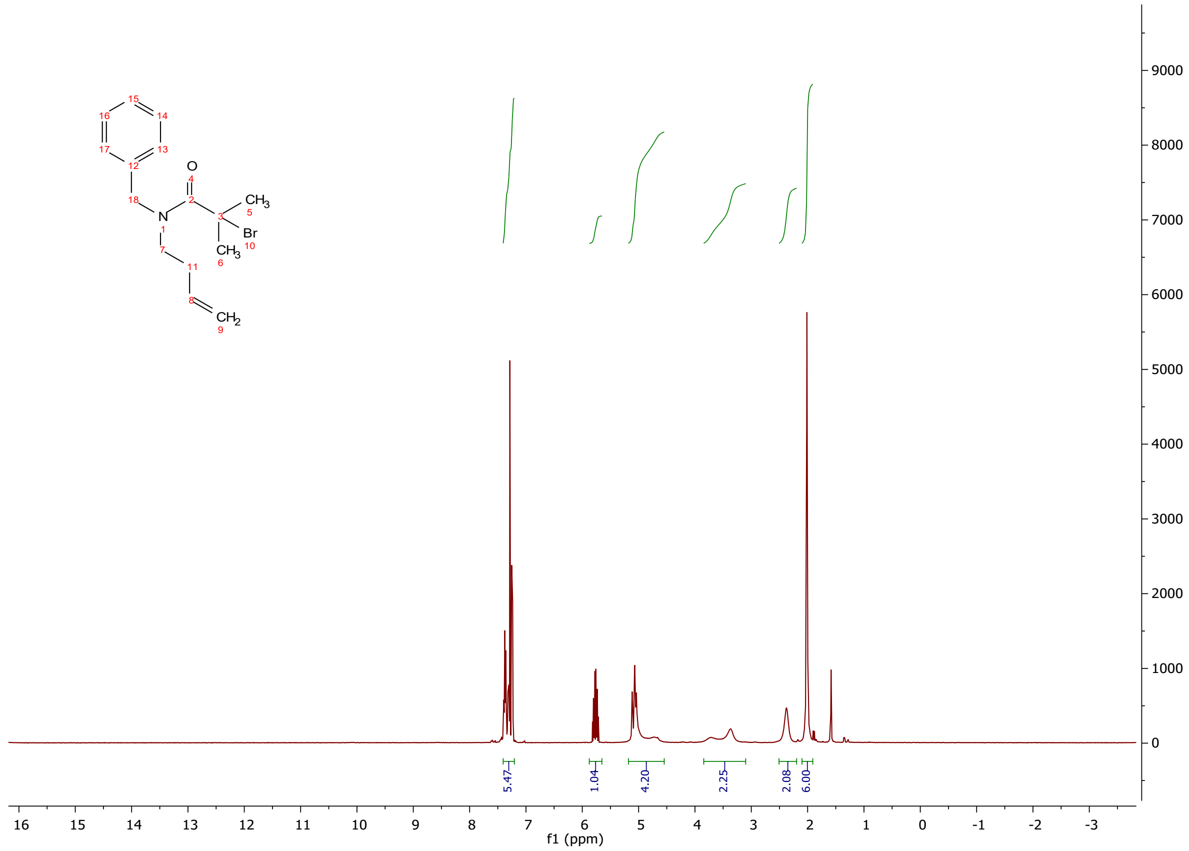

1H NMR spectra of 1-benzyl-4-(bromomethyl)-3,3-dimethylpiperidin-2-one (**8`**) :

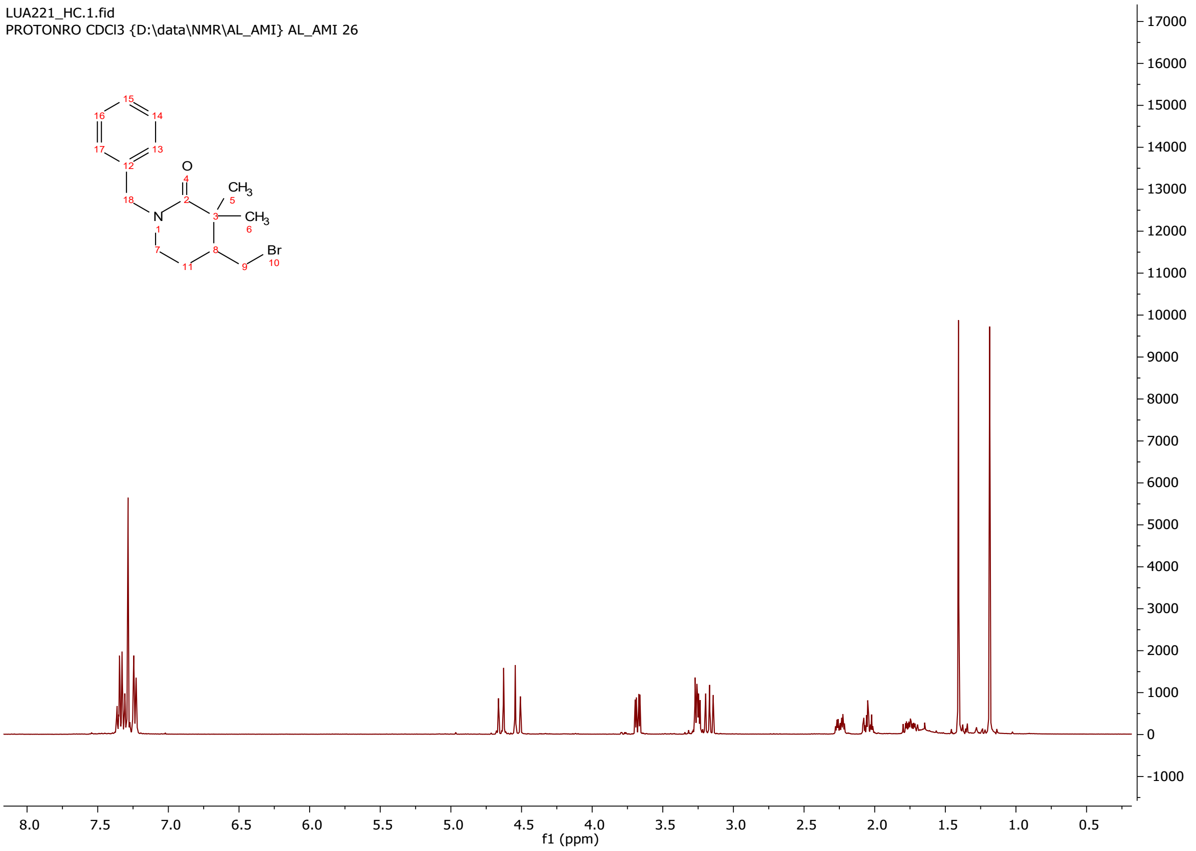

13C NMR spectra of 1-benzyl-4-(bromomethyl)-3,3-dimethylpiperidin-2-one (**8`**) :

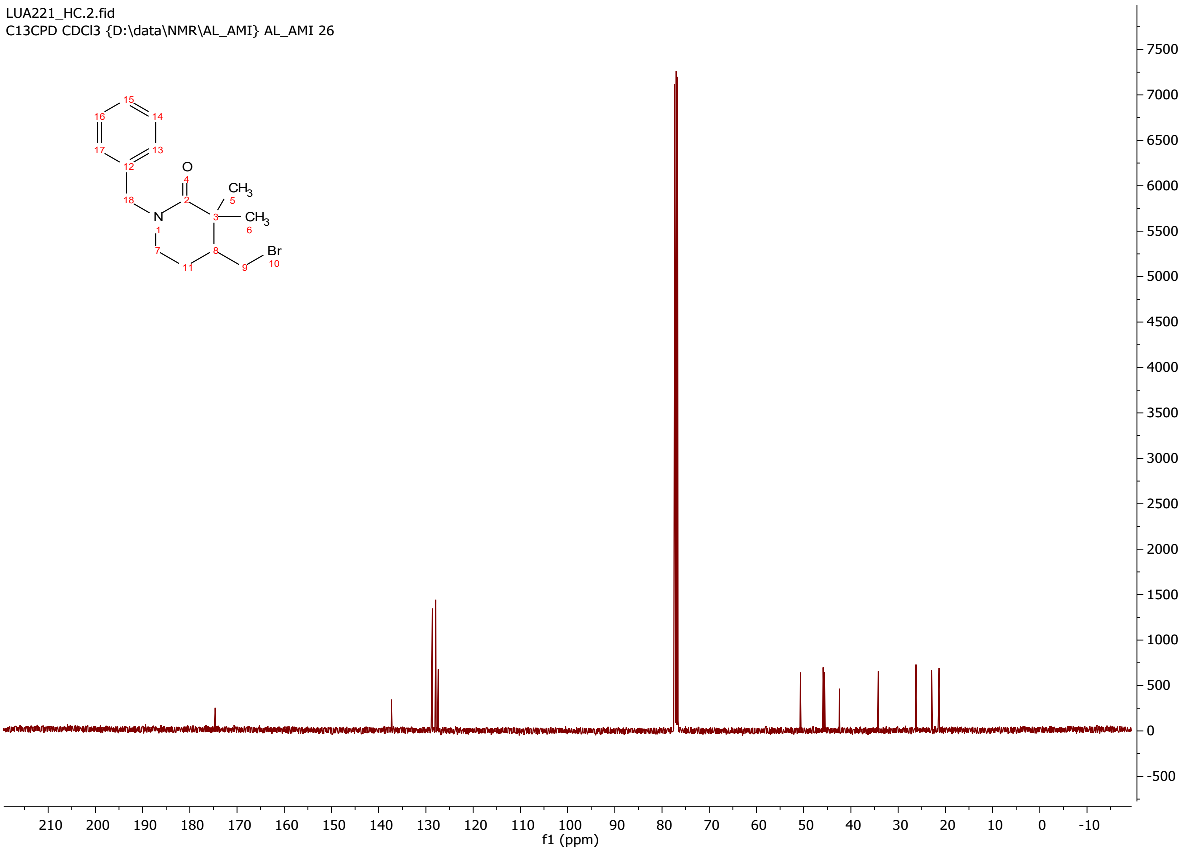

References

[1] I. Barr, F. Guo, *Bio-protocol* **2015**, *5*, e1594.

[2] I. Efimov, G. Parkin, E. S. Millett, J. Glenday, C. K. Chan, H. Weedon, H. Randhawa, J. Basran, E. L. Raven, *FEBS Lett.* **2014**, *588*, 701-704.

[3] A. J. Clark, A. E. C. Collis, D. J. Fox, L. L. Halliwell, N. James, R. K. O'Reilly, H. Parekh, A. Ross, A. B. Sellars, H. Willcock, P. Wilson, *The Journal of Organic Chemistry* **2012**, *77*, 6778-6788.

[4] Y. Motoyama, K. Kamo, A. Yuasa, H. Nagashima, *Chem. Commun.* **2010**, *46*, 2256-2258.

[5] D. Kaiser, V. Tona, C. R. Goncalves, S. Shaaban, A. Oppedisano, N. Maulide, *Angew. Chem. Int. Ed. Engl.* **2019**, *58*, 14639-14643.

[6] Q. Zhou, M. Chin, Y. Fu, P. Liu, Y. Yang, *Science* **2021**, *374*, 1612-1616.
